# The first crystal structures of hybrid and parallel four-tetrad intramolecular G-quadruplexes

**DOI:** 10.1093/nar/gkac091

**Published:** 2022-02-25

**Authors:** Dana Beseiso, Erin V Chen, Sawyer E McCarthy, Kailey N Martin, Elizabeth P Gallagher, Joanne Miao, Liliya A Yatsunyk

**Affiliations:** Department of Chemistry and Biochemistry, Swarthmore College, 500 College Ave, Swarthmore, PA 19081, USA; Department of Chemistry and Biochemistry, Swarthmore College, 500 College Ave, Swarthmore, PA 19081, USA; Department of Chemistry and Biochemistry, Swarthmore College, 500 College Ave, Swarthmore, PA 19081, USA; Department of Chemistry and Biochemistry, Swarthmore College, 500 College Ave, Swarthmore, PA 19081, USA; Department of Chemistry and Biochemistry, Swarthmore College, 500 College Ave, Swarthmore, PA 19081, USA; Department of Chemistry and Biochemistry, Swarthmore College, 500 College Ave, Swarthmore, PA 19081, USA; Department of Chemistry and Biochemistry, Swarthmore College, 500 College Ave, Swarthmore, PA 19081, USA

## Abstract

G-quadruplexes (GQs) are non-canonical DNA structures composed of stacks of stabilized G-tetrads. GQs play an important role in a variety of biological processes and may form at telomeres and oncogene promoters among other genomic locations. Here, we investigate nine variants of telomeric DNA from *Tetrahymena thermophila* with the repeat (TTGGGG)_n_. Biophysical data indicate that the sequences fold into stable four-tetrad GQs which adopt multiple conformations according to native PAGE. Excitingly, we solved the crystal structure of two variants, TET25 and TET26. The two variants differ by the presence of a 3′-T yet adopt different GQ conformations. TET25 forms a hybrid [3 + 1] GQ and exhibits a rare 5′-top snapback feature. Consequently, TET25 contains four loops: three lateral (TT, TT, and GTT) and one propeller (TT). TET26 folds into a parallel GQ with three TT propeller loops. To the best of our knowledge, TET25 and TET26 are the first reported hybrid and parallel four-tetrad unimolecular GQ structures. The results presented here expand the repertoire of available GQ structures and provide insight into the intricacy and plasticity of the 3D architecture adopted by telomeric repeats from *T. thermophila* and GQs in general.

## INTRODUCTION

G-quadruplexes (GQs) are non-canonical DNA structures composed of stacks of guanine tetrads (G-tetrads) maintained by π–π interactions. Each G-tetrad consists of four guanines in a square planar arrangement bonded via eight Hoogsteen hydrogen bonds. Monovalent cations, notably K^+^ or Na^+^, bring further stability to the overall architecture; the formation of GQs in the presence of other cations such as NH_4_^+^, Rb^+^, Cs^+^, Sr^2+^ and Tl^+^ has also been explored (1). GQs, unlike their dsDNA counterparts, are highly polymorphic. Their structures can be classified in terms of stoichiometry (uni-, bi-, tri- or tetramolecular) and strand orientation (parallel, antiparallel and hybrid). Bases other than Gs can be part of the G-tetrad, and bulges can be formed in addition to loops (2). GQ topology depends on the DNA sequence and length, the nature of the monovalent cation, the presence of ligands or additives, and the annealing conditions. Within a GQ, nucleotides connecting the G-tetrads, known as loops, can alter the conformation and stability of the structure adding an additional dimension to GQ diversity (3–5).

A high resolution sequencing method known as G4-seq identified over 700,000 sequences with G-quadruplex forming potential in the human genome (6,7) within telomeres, oncogenic promoters, and untranslated regions of mRNA (8). Telomeres are repetitive, G-rich DNA sequences that exist at the ends of linear chromosomes and aid in their stabilization and protection (9). With each cell cycle, telomeres shorten progressively, limiting the cell life span. The upregulation of the enzyme telomerase allows for the continuous extension of telomeres leading to cell immortality and uncontrolled division, which are the hallmarks of cancer (9). Telomerase was capable of extending Na^+^-stabilized intermolecular GQs due to their rapid unfolding (10). In addition, telomerase was able to bind K^+^-stabilized intermolecular GQs. However, the formation of robust K^+^-stabilized intramolecular GQs successfully inhibited telomerase activity (9,10) which suggests that GQs can be utilized as targets for the development of anticancer therapeutics.

In this work, we focus on the telomeric repeat from *Tetrahymena thermophila* (referred to as TET) which has the overall sequence (TTGGGG)_*n*_. *T. thermophila* is a unicellular eukaryotic holotrichous ciliate usually found in freshwater (11). The folding of its four repeat (TTGGGG)_4_ into a stable GQ was first demonstrated in 2011 by the Mergny laboratory (12). Parallel telomeric GQs from *T. thermophila* have been found to bind Lia3, a protein which regulates the accuracy of DNA elimination events at G-rich sequences (13), suggesting the involvement of GQs in the regulation of chromosomal organization. TET telomeric repeats differ from human telomeric repeats, (TTAGGG)_*n*_, by a point mutation (underlined), but their GQs are notably different in topology and structure. Due to a lower guanine content, human telomeric GQs are less thermally stable than TET GQs (14). In addition, TET GQs are found to be more prone to mutations than human GQs (14). Our interest in the (TTGGGG)_*n*_ DNA sequence and its structure also stems from research on an HIV aptamer with the phosphorothioate sequence TTGGGGTT. This aptamer forms a parallel tetramolecular four-tetrad GQ. Interaction of this GQ with the gp120 V3 loop in the HIV envelope prevents virions from binding to cells inhibiting virus transmittance (15). Structural information on this sequence can therefore inform the development of novel HIV therapies.

Several telomeric TET GQ structures have been reported to adopt antiparallel, parallel, and hybrid topologies demonstrating a high structural diversity of this DNA motif (16–21). Specifically, the sequence d(TGGGGT)_2_ in 100 mM Na^+^ forms two interconverting asymmetric bimolecular GQs. Both GQs adopt an antiparallel conformation with four G-tetrads and two TT lateral loops (16). NMR and crystal structures of short, single repeat variants d(TTGGGG), d(TTGGGGT) and d(TGGGGT) reveal a four G-tetrad parallel GQ conformation in the presence of K^+^ or Na^+^ (17,19–21). Finally, d(TTGGGG)_4_ in 100 mM Na^+^ forms a hybrid unimolecular GQ consisting of three G-tetrads, two lateral loops (GTTG and TTG), one propeller loop (TT), and 5′-TT and 3′-G overhangs (18). Therefore, there is a need for greater clarity in understanding how the length of *T. thermophila*’s telomeric repeats and its exact composition affect the resulting GQ structure.

In this paper, we report on the biophysical characterization of nine TET variants for their conformations, stability, and homogeneity. Furthermore, we report four high-resolution K^+^-stabilized crystal structures of two variants, TET25 (one structure) and TET26 (three structures). TET25 crystallized in a hybrid conformation, while TET26 crystallized in a parallel conformation, thereby confirming an unusual structural plasticity of *T. thermophila* telomeres. To our knowledge, these are the first hybrid and parallel four-tetrad unimolecular GQ structures. Thus, the work presented here provides further insight into the diversity of GQ structures aiding in their utilization as targets for anticancer or antiviral therapeutics.

## MATERIALS AND METHODS

### DNA and buffers

All DNA sequences were purchased from Integrated DNA Technology (IDT), hydrated to 1 or 2 mM in ddH_2_O, and stored at 4 or −20°C. DNA concentration was measured at 95°C using the extinction coefficients listed in Supplementary Table S1. A 10K buffer consisting of 10 mM lithium cacodylate pH 7.2 and 10 mM KCl (ionic strength of 20 mM) was used in this work. To promote homogeneous GQ folding, we tested three annealing protocols. The first protocol, being the simplest, consisted of annealing DNA in 10K buffer at 95°C for 5 min, cooling the samples for ∼4 h to room temperature, and equilibrating at 4°C overnight. In the second protocol, DNA was heated in 10 mM lithium cacodylate pH 7.2 at 95°C for 2 min; 10 mM KCl was then added, and the samples were heated for additional 3 min, cooled for ∼4 hours, and equilibrated at 4°C overnight. In the third protocol, we utilized LiOH treatment (22) which is known to prevent formation of thermodynamically trapped GQs and higher order oligomers. DNA samples were prepared in water, treated with 100 mM LiOH for 15 min at 39°C, and neutralized with HCl. The samples were diluted to the desired volume using a combination of 10 × 10K buffer and water (such that the resulting buffer is 10K), annealed at 95°C for 5 min, cooled for ∼4 h, and equilibrated at 4°C overnight. The purity and homogeneity of all samples were tested via PAGE, Supplementary Figure S1A. The addition of KCl during the annealing step is simple and efficient at eliminating higher order species. Thus, the second protocol was used to prepare all TET samples.

### UV-vis spectroscopy

A Varian Cary 300 UV-vis spectrophotometer equipped with an Agilent Technologies temperature controller was used to collect all UV–vis data (±0.3°C error). DNA absorbance was collected from 220 to 349 nm in quartz cuvettes with a 1 cm pathlength. Collection parameters consisted of 0.5 nm intervals, 0.1 s averaging time, 300 nm/min scan rate, 2 nm spectral bandwidth, and automatic baseline correction.

#### Concentration dependence of melting transition using UV–vis melt

Five DNA samples were prepared at 5, 50, 100, 200 and 600 μM in 10K buffer. The samples were placed into 1 cm, 2 mm, 1 mm and 0.11 mm cuvettes to maintain Abs near 1.0. UV–vis melting experiments were conducted from 20 to 90°C with 1°C step, 0.4°C/min temperature rate, 2.00 s averaging time, and 2.00 nm spectral bandwidth. Absorbance was monitored at 295 and 335 nm. DNA does not absorb at 335 nm, therefore any signal at this wavelength was due to instrument error and was used to correct the absorbance at 295 nm. The rest of the data processing followed the protocol for CD melt (see below).

#### Thermal difference spectra (TDS)

UV–vis scans taken at 20°C (where the DNA is folded) were subtracted from scans taken at 95°C (where the DNA is unfolded) which were obtained after 10 min of equilibration. The observed absorbance difference reflects the contribution of base interactions and thus the DNA secondary structure. A trough at 295 nm and two peaks at 273 and 240 nm are characteristic of a GQ topology (25). All TDS spectra were zeroed and divided by the respective DNA concentration to represent the absorbance difference per [GQ].

### Circular dichroism

All CD experiments were performed using an Aviv 435 circular dichroism spectrophotometer equipped with a Peltier thermocontroller (±0.3°C error) in 1 cm quartz cuvettes.

#### CD scan

CD scans were collected on 3–7 μM DNA samples annealed in 10K buffer. Five to seven CD scans were collected at 20°C from 220 to 330 nm with 1 s averaging time, 2 nm bandwidth and 1 nm step. CD data were processed as described in our earlier work (26).

#### CD melt

We monitored the wavelength at the maximum CD signal, either 264 or 294 nm, while varying the temperature from 20 to 95°C. Other parameters were as follows: a 1°C step, 1°C/min temperature rate, 15 s averaging time, and 5 s equilibration time. Reverse scans were also collected to calculate the hysteresis, Δ*T*_m_, defined as the difference in melting temperatures determined from melting and cooling curves. Considering the high hysteresis of TET GQs caused by sample heterogeneity (6–11°C; see Result section for details), we report the temperatures at half transition (*T*_1/2_) in place of *T*_m_. *T*_1/2_ values were calculated by taking the first derivative of the melting or cooling curves, smoothing the derivatives using a 13-point Savitzky-Golay quadratic function, and visually reading off the temperature at the peak or trough. *T*_1/2_ values are only reproducible under the same experimental conditions (e.g. the same buffer and temperature change rate) and represent a point at which half of the species present in solution are unfolded. Reported *T*_1/2_ values represent the average of two to four trials. Representative CD melting curves are shown in Supplementary Figure S2. Data processing was done using Origin 9.1 Software.

### Native polyacrylamide gel electrophoresis (PAGE)

DNA samples prepared at ∼110–240 μM in 10K buffer were loaded on 15% gels prepared with 10 mM KCl and 1× Tris–borate-EDTA running buffers. All DNA samples contained 7% sucrose. Gels were premigrated for 30 min at 150 V before 6 μl of each sample was loaded and run for 120 min at 150 V. A ladder of dT_15_, dT_24_, dT_30_, dT_57_ and dT_90_ was used as a length marker. Additionally, **T1** DNA was used as a reference for dimeric parallel GQ; **T7** was used as a reference unimolecular parallel GQ; and **19wt** DNA was used as a reference for unimolecular antiparallel GQ. The gel was visualized with Stains-all and imaged using a conventional scanner or a smart phone camera.

For concentration gels, DNA samples were annealed at the desired concentration (5, 50, 100, 200 and 600 μM) and loaded such that in all cases but 5 μM, equal amount of DNA was placed on the gel for the ease of comparison. For gels that contained crystals, three samples were loaded: (i) sample prepared at 100 μM concentration; (ii) 5–10 crystals harvested and washed three times in the mother liquor and dissolved in 10K buffer and (iii) a sample used to crystallize the DNA. Raw PAGE images for all gels are shown in Supplementary Figure S15.

### X-ray crystallography

All variants were initially screened using two commercial screens: Helix (Molecular Dimensions) (27) and Natrix (Hampton Research), and an in-lab made screen: Amber (28). Crystallization trays of 96 wells were set by a TTP Labtech Mosquito Crystal robot (equipped with a humidity chamber) using the hanging drop vapor diffusion method. Each drop contained 0.2 μl of DNA and 0.2 μl of the screen condition. The wells contained 100 μl of the screen conditions. Each crystal hit was optimized manually in 24-wells trays. We successfully obtained diffraction-quality crystals of TET25 and TET26. Diffraction data were collected at the Advanced Photon Source (beamlines 24 ID-C and ID-E) to a maximum resolution of 1.56 Å for TET25 and 1.99, 1.97, and 2.00 Å for three crystal forms of TET26.

#### Crystallization of TET25

For crystallization trials, TET25 samples were annealed at 1.0 mM in 10K buffer. TET25 crystals were initially found in Natrix 2–18 condition containing 45% MPD, 0.08 M KCl, 0.02 M MgCl_2_, 0.04 M sodium cacodylate buffer at pH 6.0, and 0.012 M spermine tetrahydrochloride. Only MPD and KCl were optimized to 39% MPD and 0.165 M KCl, yielding long thin rod-shaped crystals within a few days. Crystals were harvested without additional cryoprotection and flash frozen in liquid nitrogen.

#### Crystallization of TET26

For crystallization trials, TET26 samples were annealed at 1.5 mM in 10K buffer and in 10 mM sodium cacodylate buffer pH 6.5 with 10 mM KCl. The latter buffer yielded crystals with higher quality diffraction pattern (in general, the presence of KCl overwrites the effect of both Li^+^ and Na^+^ ions on GQ fold. In addition, pH does not have a large effect on GQ folding (29)). TET26 crystals were initially found in Amber 1–24 condition containing 30% PEG 2000, 0.3 M NaCl, and 0.05 M sodium cacodylate buffer at pH 6.5. Only PEG 2000 was optimized to 32% yielding irregular clamshell shaped crystals within a few days. Crystals were harvested and cryoprotected using the well solution, to which we added 10% ethylene glycol.

#### Molecular replacement

Diffraction data were processed in RAPD software provided by the beamline. All structures were solved by molecular replacement (MR) using Phenix (30). GQ models were obtained from the Protein Data Bank (PDB) (31).

**TET25** was solved in *P*12_1_1 space group using *Oxytricha* telomeric GQ as a model (PDB ID: 1JRN). The model included a GQ core without loops, a thymine on each 5′ and 3′ end of the four DNA strands, and three central K^+^ ions. An initial MR solution was refined and improved through extensive manual model building cycles in Coot (32) followed by Phenix Refine. The asymmetric unit (ASU) of TET25 consists of four unimolecular DNA chains (A–D), two spermine molecules, six [Mg(H_2_O)_6_]^2+^ ions, and 379 waters. Chain B was modeled in two alternative conformations, BA and BB.

**TET26-1** was solved in *P*3_1_21 space group using the GQ core and ions of the parallel tetra-molecular GQ with the sequence TGGGGT (PDB ID: 1O0K). The Na^+^ ions of the structure were replaced with K^+^ prior to performing MR. The ASU of TET26-1 consists of one DNA chain (A) that forms a single unimolecular GQ with three K^+^ ions located between the G-tetrads, a sodium ion located near the first tetrad, and 30 water molecules. The GQ in the ASU forms a dimer with a symmetry generated GQ. The two monomers within the dimer are offset such that the channels of K^+^ ions do not line up.

**TET26-2** was solved in *P*2_1_2_1_2 using the TET26-1 structure as a model with its GTT overhang removed. The ASU of TET26-2 consists of one DNA chain (A) that forms a single unimolecular GQ which creates a perfectly aligned dimer with a symmetry generated GQ. The structure includes three K^+^ ions located between the G-tetrads, a fourth K^+^ ion located at the dimer interface (on the special position), and nine water molecules. The perfect dimerization results in one long ion channel containing seven K^+^ ions (as is the case for TET26-3 below). The bases for T8, T15 and T20 were not built because the electron density is either not well-defined (T8, T20) or appears in multiple places (T15) suggesting high conformational flexibility.

**TET26-3** was solved in *P*2_1_2_1_2 using the TET26-2 structure as a model. The ASU of TET26-3 consists of one DNA chain (A) that forms a single unimolecular GQ which creates a perfectly aligned dimer with a symmetry generated GQ. The structure includes three K^+^ ions located between the G-tetrads, a fourth K^+^ ion at the dimer interface (on the special position), and five water molecules. G1 in the overhang was not built due to a lack of electron density. The density around T9 and T15 was weak but visible, thus these nucleotides were built.

Data collection and refinement statistics are presented in Table 2. The identity of K^+^, Na^+^ and Mg^2+^ in TET25 and TET26 structures was confirmed using CheckMyMetal (33). Representative crystal morphologies and crystal packing for TET25 and TET26 are shown in Supplementary Figure S3.

#### Analysis of crystallographic data: G-tetrad planarity, helical twist, torsional angles, RMSD, distances, groove widths and B-factors

G-tetrad planarity and DNA backbone torsional angles were calculated following the methods described in our previous work (23). Root mean square deviation (RMSD) was calculated in PyMOL (with no outlier rejection) by pairwise alignment of the selected structures or their parts (e.g. G-quadruplex core without overhangs or without loops). Groove widths and helical twists for TET25 were determined using the program Advanced Structural Characteristics of G-quadruplexes ACS-G4 (http://tiny.cc/ascG4). Groove widths represent the distances between C3′–C3′ sugar atoms. Helical twist is defined as the average angle between the vectors that pass through C1′ atoms of two adjacent guanines in two stacked G-tetrads. Distances between adjacent G-tetrads were calculated using the centroid of each G-tetrad with an in-lab MATLAB script. Out-of-plane deviation (*D*_OOP_) was calculated via singular value decomposition with an in-lab MATLAB script. B-factors were calculated manually and with Baverage in CCP4i (34,35).

## RESULTS AND DISCUSSION

In this work, we set out to characterize the telomeric region of *Tetrahymena thermophila* composed of the repeat sequence (TTGGGG)_*n*_. We designed nine variants (Table 1) and tested their ability to fold into stable GQ structures in the presence of 10 mM K^+^ (10K buffer). Moreover, we determined the crystal structure of TET25 and three crystal forms of TET26.

**Table 1.** DNA sequences studied in this work, and their physical and thermodynamic parameters in 10K buffer. Other sequences used as controls are also included. G-rich stretches are in bold

DNA	Sequence	*T*_1/2_,°C	Hysteresis,°C	Conformation*
**TET12**	T**GGGG**TT**GGGG**T	75.1 ± 0.9	Irreversible	P
**TET14**	GTT**GGGG**TT**GGGG**T	79.5 ± 0.5	Irreversible	P + 1
**TET22**	**GGGG**TT**GGGG**TT**GGGG**TT**GGGG**	76.1 ± 0.8	7 ± 2	A^#^
**TET22A**	T**GGGG**TT**GGGG**TT**GGGG**TT**GGG**	62.2 ± 0.2	2.3 ± 0.4	H
**TET24**	TT**GGGG**TT**GGGG**TT**GGGG**TT**GGGG**	**	**	P + 2
**TET24A**	T**GGGG**TT**GGGG**TT**GGGG**TT**GGGG**T	73.6 ± 1.4	9 ± 2	P + 2
**TET25**	GTT**GGGG**TT**GGGG**TT**GGGG**TT**GGGG**	75.5 ± 0.7	5.9 ± 0.1	H + P + 1
**TET26**	GTT**GGGG**TT**GGGG**TT**GGGG**TT**GGGG**T	74.4 ± 0.4	8.1 ± 0.6	P + 1
**TET26A**	TT**GGGG**TT**GGGG**TT**GGGG**TT**GGGG**TT	73.7 ± 0.8	11 ± 1	P + 2
**T1**	**GGG**TT**GGG**TT**GGG**TT**GGG**	57.7 ± 0.3^%^	3.3	P, dimer
**T7**	T**GGG**TT**GGG**TT**GGG**TT**GGG**T	52.0 ± 0.3^%^	2.2	P, monomer
**19wt**	**GGGGG**A**GGGG**TACA**GGGGG**TACA**GGGGG**	76.9 ± 0.5^%^	-	A

*P, H and A represent Parallel, Hybrid, and Antiparallel conformations. P + 1 represents two different conformations where one is parallel. P + 2 represents three different conformations where one is parallel.

^#^In addition to the antiparallel GQ, this variant also forms a parallel dimer.

**Poorly defined melting transition.

% data from ref (23) for **T1** and **T7** and from ref (24) for **19wt** in 5K buffer (5 mM KCl, 95 mM LiCl and 10 mM lithium cacodylate 7.2).

### Design of the *T. thermophila* variants

Nine variants of the *T. thermophila* telomeric repeats were designed based on either sequences previously characterized via NMR (18) and biophysical methods (10,36), or by extending the minimum sequence (GGGGTT)_3_GGGG in the 5′ or 3′ directions in accordance with its genomic context by 5′-T, 5′-TT and 5′-GTT as well as 3′-T and 3′-TT in different permutations (Table 1). The sequences are numbered based on their length from TET12 to TET26A. The A label is assigned arbitrarily to distinguish sequences with the same length.

TET12 and TET14 contain two G-rich stretches and are expected to form bimolecular GQs. TET22A has three GGGG stretches and one GGG stretch and is expected to form an intramolecular GQ with only three G-tetrads. All other GQs have four GGGG stretches and are expected to form intramolecular GQs with four G-tetrads. All sequences examined are biologically relevant (i.e. no mutations).

### Biophysical characterization of TET sequences

**Figure 1.**
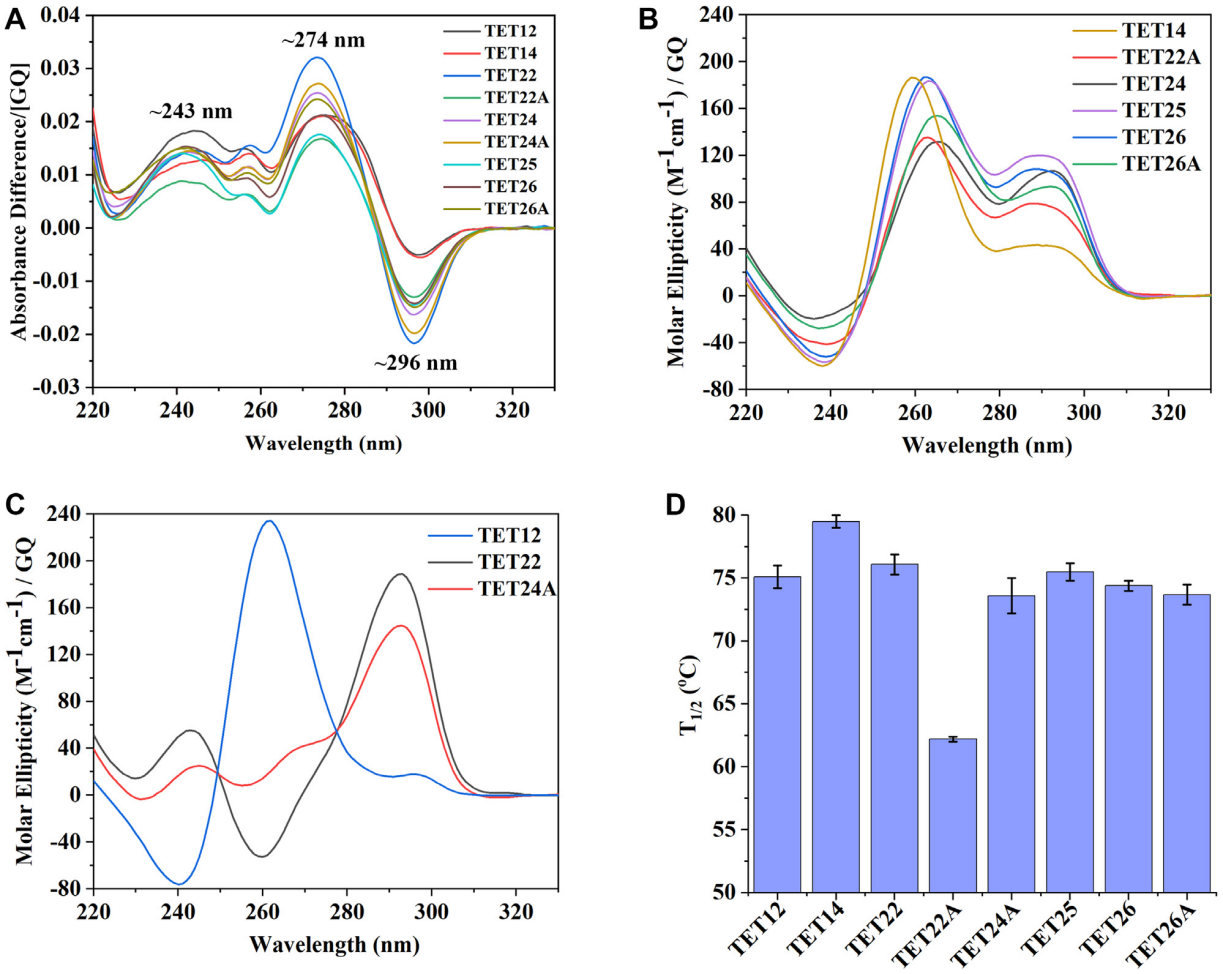
Biophysical characterization of TET sequences. (**A**) TDS. (**B**, **C**) CD scans at 20°C. (**D**) Thermal stability determined via CD melts. All samples were prepared in a 10K buffer at ∼4 μM per GQ.

As a first step of TET DNA characterization, we employed several biophysical methods, such as TDS, CD scan, thermal melt, and native PAGE. The TDS signature of all TET variants in 10K buffer (Figure 1A) contains the characteristic trough at ∼296 nm and peaks at ∼243 and ∼274 nm confirming GQ secondary structure (25).

Once GQ fold was established via TDS, we analyzed CD scans to determine the type of GQ structure—parallel, antiparallel or hybrid. Typically, parallel GQs exhibit a peak at ∼264 nm and a trough at ∼240 nm, antiparallel GQs exhibit a peak at ∼295 nm and a trough at ∼260 nm, and hybrid conformations exhibit both ∼264 and ∼295 nm peaks in addition to a ∼240 trough (37). CD scans reveal a hybrid signal for most sequences with one peak at 263–265 nm and another at ∼290 nm, Figure 1B. On the other hand, TET22 and TET24A form predominantly antiparallel structures with a major peak at 293 nm, Figure 1C. However, a small or absent trough at 260 nm suggests the presence of minor hybrid or parallel components. Consistent with this data, a previous study showed that TET22 at 3 μM in the presence of 100 mM KCl displays an antiparallel CD signal with some parallel component that increases with increasing strand concentration (to 10 and 30 μM) (12). Finally, TET12 forms a parallel conformation with a major peak at 262 nm and a trough at 240 nm, Figure 1C. Combined, CD and TDS data indicate that all examined TET sequences form GQ structures with a variety of possible conformations.

Next, we assessed the stability of TET GQs via CD melting experiments (see Supplementary Figure S2 for representative melting curves). All TET sequences but TET22A display a significant hysteresis of ∼6–11°C suggesting either the presence of multiple species or a folding pathway that includes intermediates. The melting of TET12 and TET14 is irreversible due to the slow folding kinetics of four G-tetrad bimolecular GQs. High hysteresis is the reason that we do not report *T*_m_ values (T_m_ is a thermodynamic parameter characterizing a specific secondary structure). Rather, we report temperature at half transition, *T*_1/2_, which characterizes the given sample (in our case a mixture of 2–3 GQ conformations, see PAGE below) under given conditions (e.g. buffer and temperature change rate). The summary of T_1/2_ values is given in Figure 1D and Table 1. All structures display high and similar stability (T_1/2_= 73.6 - 79.5°C) except TET22A whose T_1/2_ is significantly lower, 62.2°C, likely because it is predicted to form a three G-tetrad GQ. This finding not only emphasizes the importance of the number of G-tetrads to GQ stability, but also supports the presence of four G-tetrads in the remaining TET variants. An earlier study of TET22 in 100 mM KCl reported its stability to be >80°C (12), consistent with our value of 76.4°C in 10K buffer when the effect of potassium on GQ stability is considered. Specifically, in our earlier work we demonstrated that the stability of another G-rich sequence, **T1**, increased from 65.6°C in 20 mM K^+^ buffer to 76.7°C in 100 mM K^+^ buffer (23). The fact that *T*_1/2_ is similar (within 5°C) for all four G-tetrad TET GQs agrees with the recent finding by Mergny and Li laboratories who discovered no correlation between conformation and stability for 99 three G-tetrad GQs (38).

### Homogeneity and molecularity of TET GQs via native PAGE

**Figure 2.**
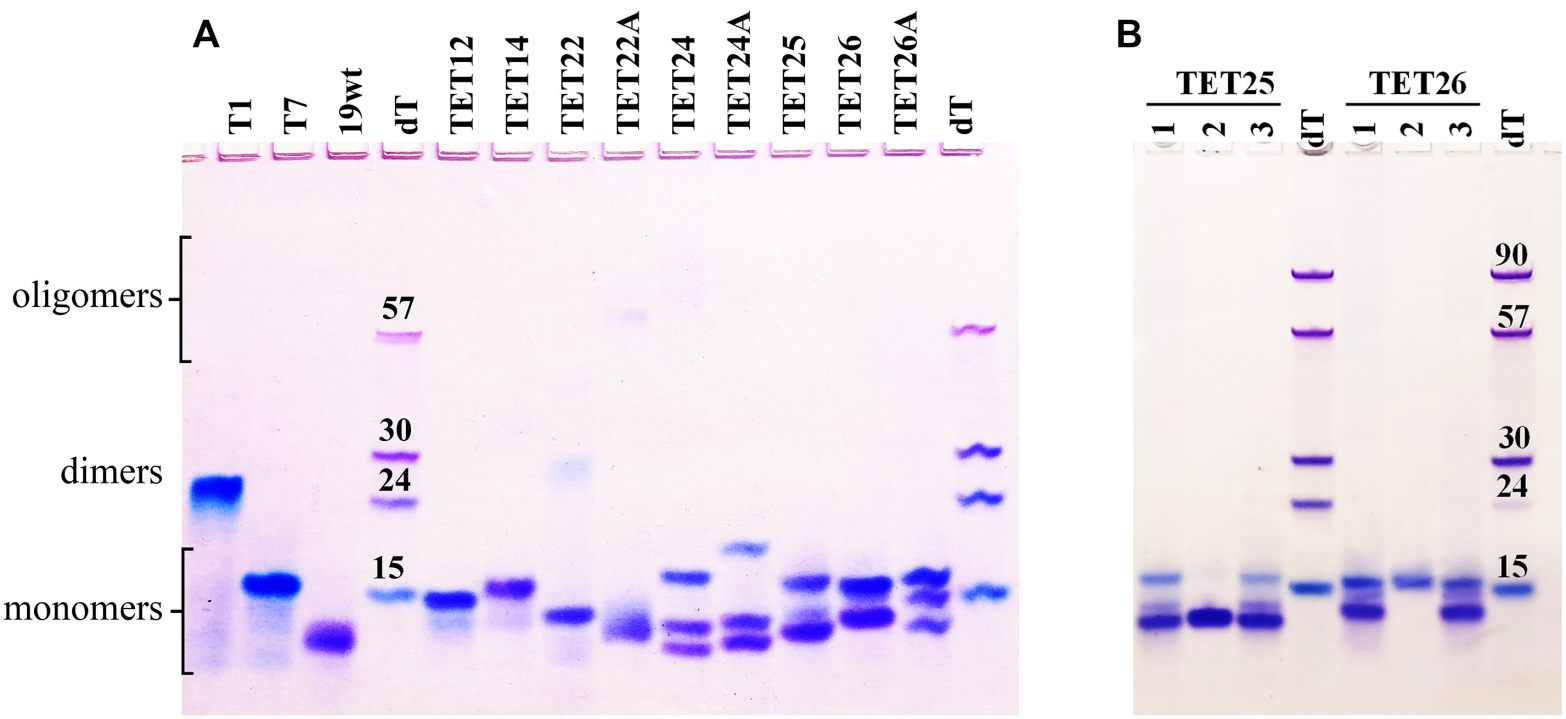
Homogeneity, molecularity, and conformation of TET GQs via native PAGE. Fifteen percent gel was prepared in 1 × TBE supplemented with 10 mM KCl. (**A**) The DNA samples were prepared in 10K buffer at ∼0.10–0.24 mM. Size markers correspond to dTn sequences. Controls include **T1**, **T7**, and **19wt**. (**B**) The DNA samples were prepared the following way: lane **1** – 0.10 mM DNA in 10K buffer; lane **2** – crystals dissolved in 10K buffer; and lane **3** – concentrated DNA sample used to grow crystals deposited in lane 2.

The results of native PAGE (Figure 2A) show that most of the TET sequences form 2–3 major bands. The presence of multiple bands on PAGE explains the hysteresis observed in the CD melting studies (Table 1). It is also consistent with the reported diversity of GQ folds adopted by *T. thermophila* telomeric DNA (16–18). The position of the major bands on PAGE indicates the formation of intramolecular or, in the case of TET12 and TET14, bimolecular GQ monomers. TET22 is the only sequence that also displays a faint slower moving band corresponding to a GQ dimer, while TET22A and TET24 display a small amount of higher order oligomers. Dimer formation for TET22 is expected as this sequence starts with a 5′ G-stretch. G-rich DNA sequences with a 5′ G-stretch frequently dimerize (23,39–41). While we observed only a small amount of the TET22 dimer at 10 mM KCl, the Mergny lab, who studied the same sequence via PAGE, observed a dimeric GQ at 100 mM KCl (12). This observation is in agreement with an NMR study which suggests that higher amounts of KCl favor dimer formation (42). To avoid dimer formation, a non-G nucleotide (adenine or thymine) needs to be placed at the 5′ end with the former being better at breaking the dimer (42). Consistent with these observations, all other TET sequences do not display any dimerization. We have confirmed the molecularity of TET GQs using three previously characterized GQ structures: a four-tetrad antiparallel monomeric **19wt** GQ (24), a three-tetrad parallel monomeric **T7** GQ, and a three-tetrad parallel dimeric **T1** GQ (23).

### Conformations of TET GQs via native PAGE

To better understand the topology of different bands present on PAGE, we resorted to two approaches. First, we employed a highly selective GQ ligand, *N*-methyl Mesoporphyrin IX (NMM), known to induce a parallel GQ conformation (26). We annealed selected TET sequences, TET25, TET26 and TET26A, with 2 eq. of NMM and observed increased homogeneity of the samples. Specifically, NMM converted multiple GQ conformations into a single parallel GQ-NMM complex according to CD and PAGE (Supplementary Figure S1A and B). The GQ-NMM complexes and parallel monomeric **T7** GQ lined up with the slower moving bands of the DNA alone on the PAGE, suggesting that those bands correspond to a parallel GQ conformation. A detailed report on the interactions between NMM and TET sequences is forthcoming.

In our second approach, we collected TET25 and TET26 crystals and visualized them on PAGE (Figure 2B). According to our crystallographic studies (see below), TET25 crystallized in a mixed-hybrid conformation, while TET26 crystallized in the parallel conformation. Using this piece of information combined with the data from the NMM study, we can conclude that the faster moving bands on PAGE typically represent mixed-hybrid or antiparallel structures, while the slower moving bands typically represent parallel GQs. The slower mobility of parallel GQs was observed earlier (26) and could be explained by their larger hydrodynamic radius due to the presence of propeller loops.

Equipped with this knowledge, we can now suggest possible conformations for each of the TET variants based on their CD signals and PAGE mobility (Figures 1B–C and 2). Bimolecular TET12 and TET14 display one major band on the gel and predominantly parallel CD signatures. Thus, both variants adopt predominantly parallel GQ conformations. The small ∼295 nm shoulder in the CD of TET14 may correspond to a small amount of antiparallel or mixed-hybrid GQ. For TET22, the major band likely corresponds to an antiparallel GQ according to its CD signature. Due to the presence of a 5′ G-stretch, this variant also forms a small amount of a (likely parallel) GQ dimer. TET22A displays a single, albeit smeary, band on PAGE. Coupled with low hysteresis in melting studies (2.3°C; Table 1), the data argue for the homogeneity of this GQ which likely adopts a hybrid conformation according to CD. Both TET24 and TET24A fold into three species according to PAGE (two faster moving bands and one slower band). We can suggest that the slower moving band corresponds to a parallel GQ. For TET24A, based on its mostly antiparallel CD signature, the two faster moving bands likely represent antiparallel GQs. The hybrid CD signature of TET25, TET26 and TET26A represents a superposition of the CD signatures for three GQ conformations. It is clear from our crystallographic and PAGE studies that the slowest moving band in each case reflects a parallel GQ, while the fastest moving band represents a mixed-hybrid conformation (Figure 2B). The latter is the major conformation of TET25. The conformations for TET variants are reported in Table 1.

### Effect of 5′ and/or 3′overhangs on the structure and stability of GQs from *T. thermophila*

After determining the thermal stability and major GQ conformations of TET sequences, we can now discuss how the composition of the DNA and length of the 5′ and 3′ overhangs in *T. thermophila* affect the resulting GQ fold and stability. If this effect is minimal, then any variant represents the *T. thermophila* telomeric region with high accuracy. If this effect is strong, then the selection of specific sequences will greatly affect the experimental outcome. This information is crucial for studying new G-rich regions when a decision needs to be made on the length and composition of a possible representative construct.

For comparison, we will focus on TET22, 24, 24A, 25, 26 and 26A. All these sequences contain a (GGGGTT)_3_GGGG core but differ by their 5′ and 3′ overhangs. The stability of all sequences is within 2.5°C of each other (73.6–76.1°C) suggesting that overhangs play a minor role in GQ stability. Addition of the 5′-TT overhang to TET22 to create TET24 or 5′-GTT overhang to create TET25 changes the mostly antiparallel CD signature of TET22 to a hybrid CD for TET24 and TET25, with a higher parallel component for the latter, Supplementary Figure S4. On the native PAGE, one band observed for TET22 becomes three for both TET24 and TET25. Introducing a 3′-T (TET25 vs. TET26) or a 3′-TT (TET24 versus TET26A) also leads to an increase in the parallel fold albeit to a lesser extent, Supplementary Figure S4. On the gel, three bands for TET25 coalesce into two for TET26, but the three bands for TET24 remain, albeit shifted in position, for TET26A. Finally, introducing 5′- and 3′-T overhangs (TET22 vs. TET24A) and 5′- and 3′-TT overhangs (TET22 vs. TET26A) leads to significant increase in the parallel CD signature, especially for the latter case, Supplementary Figure S4. In both cases, the presence of overhangs increases heterogeneity (one vs. three bands on PAGE).

In sum, our observations suggest that the addition of 5′ or 3′ overhangs leads to a greater diversity of GQ conformations and increases the parallel component of GQs adopted by the (GGGGTT)_*n*_ telomeric repeat from *T. thermophila*. The observed stability of this repeat is nearly unchanged by the presence of overhangs suggesting that the stability of GQs stems from their four-tetrad GQ core. The observed plasticity, on the other hand, can be controlled by the length and nature of the 5′- and 3′-overhangs. This unique plasticity is further illustrated in our solved crystal structures of TET25 and TET26 discussed below.

## CHARACTERIZATION OF TET25 VIA X-RAY CRYSTALLOGRAPHY

TET25 forms a mixture of three GQs in 10K buffer according to PAGE. The major GQ, represented by the fastest moving intense band on the gel, adopts a mixed-hybrid conformation, Figure 2B, while the top fainter band corresponds to a parallel GQ, Supplementary Figure S1A. Under crystallization conditions, the intensity of the 264 nm CD peak decreases, suggesting a decrease in the parallel component of the TET25 mixture, Supplementary Figure S1C, and potentially explaining why it is the mixed-hybrid structure that crystallized.

### TET25 crystals and crystal organization

TET25 produced long rectangular crystals that grew over a period of 2–5 days, Supplementary Figure S3E. The crystals belong to the *P*12_1_1 space group and diffract to 1.56 Å resolution. The ASU contains four unambiguously positioned copies of four-tetrad hybrid GQs, two spermine molecules, three K^+^ per GQ (12 K^+^ in total), and six Mg^2+^ ions, Supplementary Figure S5A. The four monomers of TET25 can be described as two sets of interlocking dimers, A–C and B–D, which point toward the center with their 3′ G-tetrads and face outward with their 5′ G-tetrads. Due to the great similarities of the four TET25 GQ copies (average RMSD of 0.8 ± 0.1 Å, Supplementary Table S2), all important structural parameters were obtained by averaging the corresponding values for each TET25 monomer.

Mg^2+^ ions play an important role in DNA and RNA structures where they stabilize electrostatic clusters of phosphates (43). Such stabilization is also observed here. Two of the six Mg^2+^ ions (Mg1 and Mg2) coordinate six water molecules in their primary coordination sphere and five phosphates each in their secondary coordination sphere. Mg2 also coordinates O2 of T15 (chain A) in its secondary coordination sphere. The remaining four Mg^2+^ ions each coordinate four water molecules and two phosphates in *cis* arrangement, Supplementary Figure S5B. Mg1–5 connect to each other via a network of water molecules and a phosphate backbone.

Two spermines in the ASU promote long range interactions between individual DNA chains. Spermine 1 bridges the phosphates of G4 and G22 from chain B to the phosphates of G4′ and G22′ from chain C′. Spermine 2 contributes to similar hydrogen bonding between chains D and A′, Supplementary Figure S5C. The use of ′ indicates a symmetry generated chain, i.e. a chain not in the specified ASU.

The ASU contains 379 structural water molecules. There are extensive and highly structured water spines along the narrow groove of TET25 GQs described in detail in our earlier report (44).

### Overall architecture of the TET25 structure

**Figure 3.**
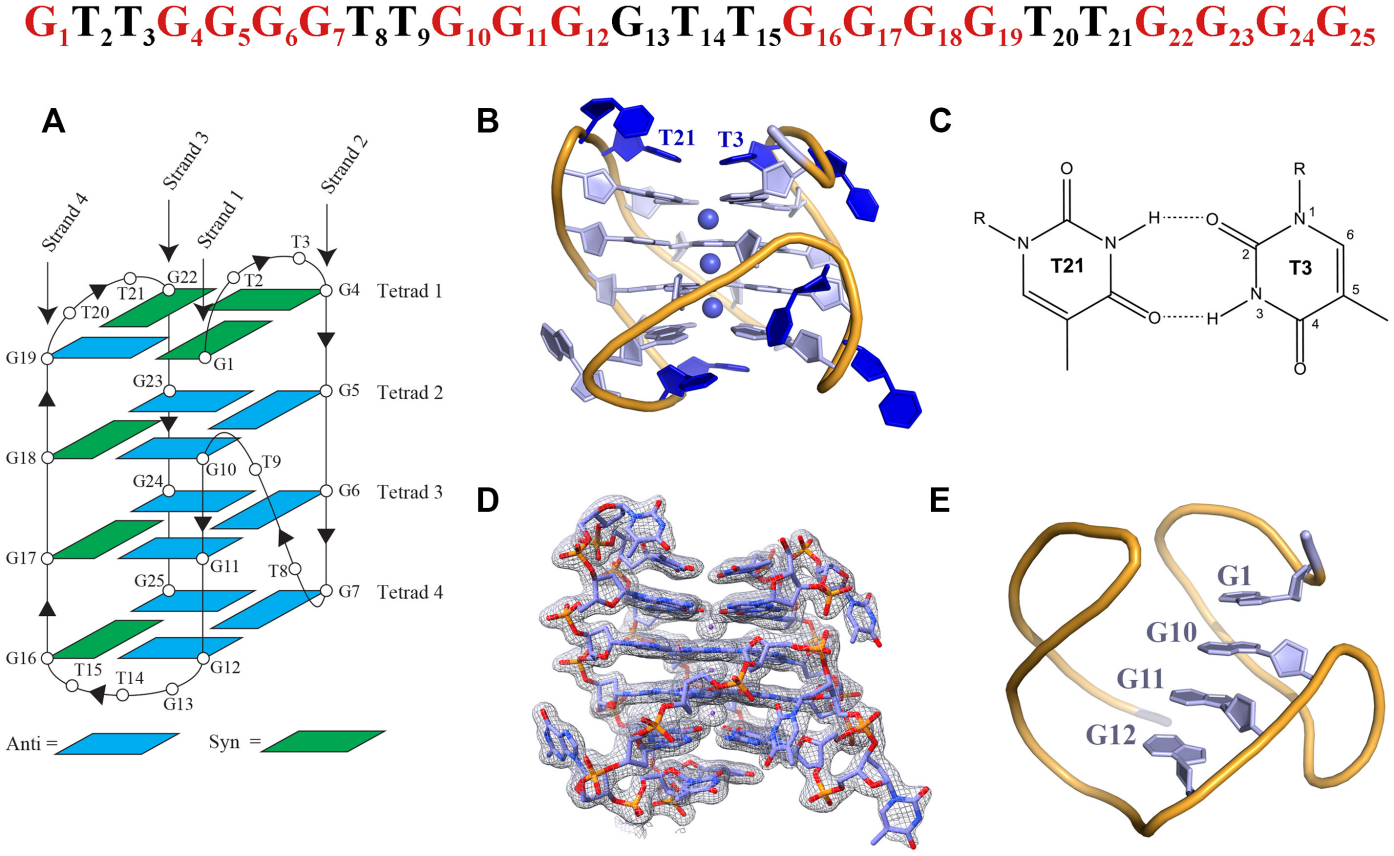
Crystal structure of the four-tetrad [3 + 1] hybrid TET25 GQ. The DNA sequence with nucleotide numbers is shown at the top. Guanines that participate in G-tetrad formation are colored in red. (**A**) Schematic representation of the folding topology with numbering schemes for nucleotides. Blue and green rectangles indicate *anti* and *syn* conformations of guanine bases, respectively. Chain orientation is indicated by arrowheads. (**B**) Cartoon representation of chain A with purines, pyrimidines, and sugars shown as filled rings and K^+^ ions as spheres. (**C**) Non-canonical T3-T21 base pair that stabilizes the GQ and maintains the position of the top lateral loops. (**D**) Chain A surrounded by the electron density at I/σ = 1.0. (**E**) 5′-top snapback.

The structure of TET25 is of high quality (*B*_av_ = 38.4 Å^2^, Supplementary Figure S6A) with all structural features clearly represented by the electron density, Figure 3. According to the GQ topology classification by Webba de Silva (45), TET25’s conformation is most similar to type V-9b, a hybrid [3 + 1] GQ. G1, G4, G16-G18 and G22 adopt *syn* glycosidic conformations, while all other guanines adopt *anti* glycosidic conformations. The most interesting aspect of the structure is the involvement of the 5′-G1(*syn*) from the 5′-GTT overhang in the top G-tetrad formation along with G19(*anti*)–G22(*syn*)–G4(*syn*). G1 displaces G13, which is part of the second GGGG stretch, into the lateral GTT loop. Therefore, in addition to two lateral loops and one propeller loop typically seen in V-9b structures, TET25 exhibits another lateral loop (overall three lateral loops and one propeller loop). The unique structural feature of a non-G-stretch guanine participating in a tetrad formation is called a snapback, in this case a 5′-top snapback, Figure 3E. The snapback structural feature was observed for the first time for *c-myc* (46) and later for *c-kit* promoter GQ DNA (47). The former can be classified as a 3′-bottom snapback, while the latter has a snapback with two middle guanines being inserted into the G-tetrad. The 5′-top snapback is rare. One example of it is in the structure with PDB ID 6KVB. The presence of a 5′-top snapback results in the propeller T8-T9 loop connecting three G-tetrads in place of four. As expected, the [3 + 1] hybrid topology of TET25 leads to one narrow (13.0 Å), two medium (∼14.2 Å), and one wide groove (15.6 Å), Supplementary Figure S7A, Table 3, and Supplementary Table S3.

Torsion angle analysis of the DNA backbone (Supplementary Figure S8A) indicates no significant outliers and is consistent with values reported for other type V GQs (45). The helical twist values are 15.8 ± 0.8°, 31.7 ± 0.6°, and 24.6 ± 0.4° between 1–2, 2–3, and 3–4 tetrads, Supplementary Table S4. These values fall into the reported range of 15–33° determined for nine selected hybrid [3 + 1] GQs (48). Out-of-plane deviations (*D*_OOP_) of tetrads 1–4 starting with the 5′ end are 1.30 ± 0.05, 1.09 ± 0.05, 1.06 ± 0.05 and 1.92 ± 0.10 Å, respectively, Supplementary Table S5, in agreement with the data collected for other [3 + 1] GQs (48). Somewhat greater planarity of the 5′ G-tetrad as compared to the 3′ G-tetrad may be explained by its π–π stacking with a non-canonical T3-T21 base pair, Figure 3B and C. At the same time, T14 and T15 stack onto 3′ G-tetrad, but because they do not form a base pair, their ability to maintain tetrad planarity is lower, Supplementary Figure S9. The tetrads in TET25 are spaced rather equally with an averaged distance of 3.43 ± 0.03 Å, Supplementary Table S6, which is the ideal distance for efficient π–π stacking (49,50).

### Loop arrangement

TET25 has three lateral loops (T2–T3, G13–T14–T15, and T20–T21) and one propeller loop (T8–T9). The propeller T8–T9 loop has T8 pointing out into the solvent and T9 resting neatly within the groove, Supplementary Figure S9A. We observed a similar general arrangement of propeller TT loops in our recently solved structures of **T1** and **T7** parallel GQs (23) as well as in TET26 structures (see below). The two lateral T2–T3 and T20–T21 loops at the 5′-end interact with each other by forming a non-canonical base pair between T3 and T21 which π stacks with the 5′ G-tetrad, Figure 3B–C and Supplementary Figure S9B. T2 and T20 nucleotides point into the solvent and along with T8 have the highest B-factors as expected due to their high flexibility, Supplementary Figure S6A. T2 displays hardly any hydrogen bonding interactions while T20 interacts with T20′ via N3–O2′ and O2–N3′ hydrogen bonds, but the two bases are not in one plane, Supplementary Figure S9E. The G13 nucleotide from the G13–T14–T15 lateral loop of chain A interlocks via π–π stacking with G13 of chain C while resting over the wide groove of chain C, Supplementary Figure S9C. Similar interactions interlock chains B and D and yield efficient packing of the monomers in the ASU. Both T14 and T15 of the GTT loop π–π stack onto the 3′ G-tetrad (Supplementary Figure S9D) such that the base of T14 π–π stacks with the base of G7 and the sugar hydrogen of T14 bonds with the base of G12. In a similar fashion, T15 π–π stacks with the base of G25 and its sugar hydrogen bonds with the base of G16. The described interactions stabilize the 3′-tetrad and the GTT lateral loop.

### K^+^ ions in the central ion channel

The central ion channel contains three K^+^ ions in a square antiprismatic coordination with eight O6 carbonyl oxygens of guanines. The middle K^+^ is equidistant from the two tetrads above and below it with an average K–O6 distance of 2.76 ± 0.06 Å. Both the 3′ and 5′-end K^+^ ions are located closer to the terminal tetrads (average distance of 2.69 ± 0.05 Å) than to the inner tetrads (average distance of 2.9 ± 0.1 Å), Supplementary Figure S10. Such uneven positioning of K^+^ is likely due to higher non-planar deformation of the terminal G-tetrads, Supplementary Table S5. The three K^+^ ions are nearly equidistant with the average distance between them of 3.50 ± 0.02 Å. Overall, the positioning of K^+^ ions is in line with the data observed for other GQ structures.

## CHARACTERIZATION OF TET26 VIA X-RAY CRYSTALLOGRAPHY

**Figure 4.**
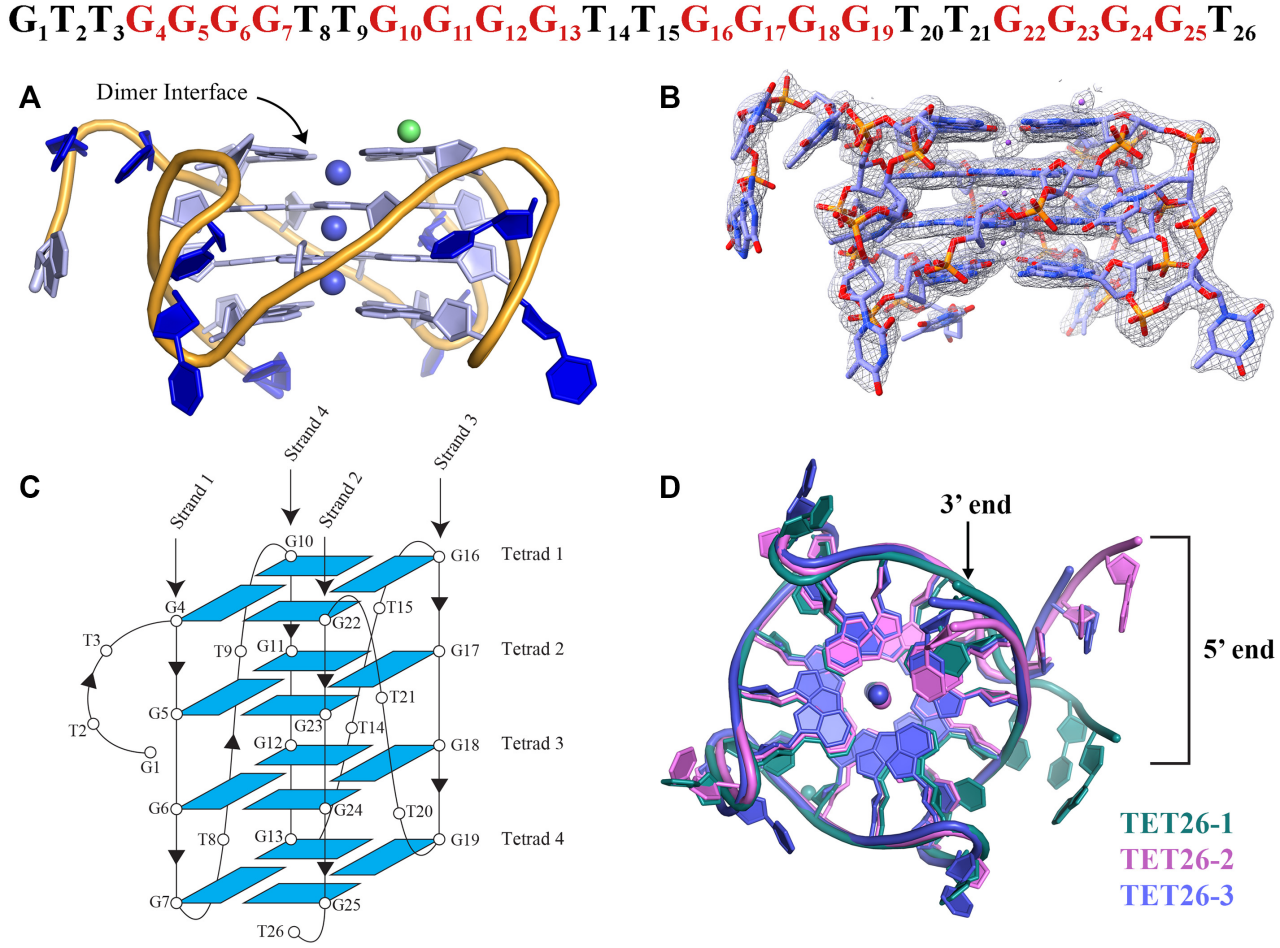
Crystal structure of TET26. (**A**) Cartoon representation of the crystal structure of TET26-1 with nucleotides and sugars shown as filled rings. Purple spheres represent K^+^ ions and a green sphere represents a Na^+^ ion. (**B**) TET26-1 surrounded by the electron density at I/σ = 1.0. (**C**) Schematic representation of the TET26 folding topology with numbering schemes for the nucleotides. All nucleotides adopt *anti* glycosidic conformation. Chain orientation is indicated by arrowheads. (**D**) Comparison of TET26-1 (green), TET26-2 (pink) and TET26-3 (purple). An overlay of the three GQ structures shows that the GQ cores and loops are nearly identical whereas the 5′-GTT and 3′-T overhangs exhibit noticeable differences.

TET26 produced irregular clamshell shaped crystals, Supplementary Figure S3E, that belong to two different space groups *P*3_1_21 (**TET26-1**) and *P*2_1_2_1_2 (**TET26-2** and **TET26-3**). The crystals diffracted to resolutions 1.99, 1.97 and 2.00 Å, respectively. According to PAGE and CD, TET26 contains nearly equal amounts of a parallel GQ and a GQ with either antiparallel or hybrid topology. PAGE gel on TET26 crystals (Figure 2B) and crystal structures of all three forms of TET26 revealed that only the parallel four-tetrad GQ crystallized. The schematics of the GQ fold with the numbering scheme for nucleotides is shown in Figure 4. As expected and contrary to what is observed for TET25, the tetrads are formed by the guanines from the four GGGG stretches. TET26 has three propeller TT loops as well as 5′-GTT and 3′-T overhangs. All guanines adopt *anti* glycosidic conformations, as is expected for parallel GQs (50). In each case, the ASU contains one GQ monomer which forms a 5′-5′ crystallization dimer with a symmetry generated partner. The monomers can be classified as type VIII-1a GQs with four medium grooves (45) of an average dimension of 14.6 ± 0.1 Å, Supplementary Table S7 and Supplementary Figure S7B. The overlay of the three TET26 structures, shown in Figure 4D, indicates a great similarity between TET26-2 and TET26-3 (RMSD of 1.862 Å), whereas TET26-1 exhibits different 5′-GTT and 3′-T overhang geometries compared to the other two structures (RMSD 3.2–3.9 Å). Setting the overhang nucleotides aside, all three TET26 structures are rather similar with RMSD of 1.266–2.007 Å, Supplementary Figure S11.

### Dimer interface and K^+^ ion channel in TET26

**Figure 5.**
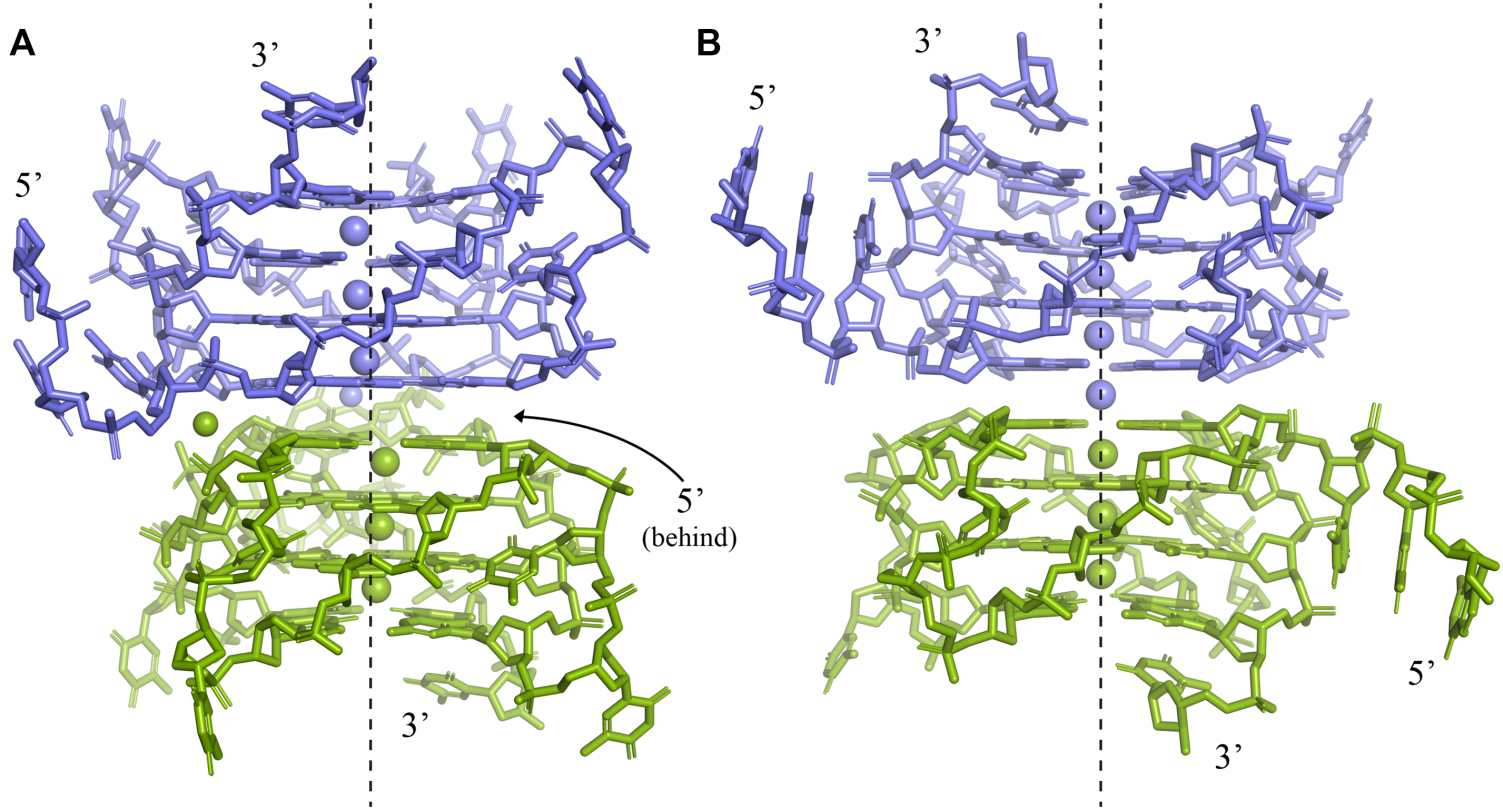
A depiction of (**A**) TET26-1 and (**B**) TET26-2 dimers with one monomer colored in purple and another in green. The symmetry related GQ was generated in PyMol. Two separate K^+^ channels exist in TET26-1. TET26-2 has one ion channel that houses seven K^+^ ions, including the K^+^ at the dimer interface which is modeled at 0.5 occupancy and colored in purple. The arrangement of TET26-3 is similar to that of TET26-2. The dashed line is drawn to guide the eye.

Among the three TET26 structures, TET26-1 is unique in that its monomers do not line up, Figure 5A, contrary to what is observed in TET26-2, -3 and in our earlier structures of parallel dimeric GQs (23,51). This offset is likely due to the stabilizing interactions surrounding TET26-1’s second loop (T14–T15) which are further detailed below and in Supplementary Figure S12. Not surprisingly given the offset, a K^+^ at the dimer interface is not observed in TET26-1. Therefore, each GQ monomer in TET26-1 contains three K^+^. On the other hand, the ion channels of TET26-2 and TET26-3 dimers contain seven K^+^ - three per each monomer and one at the dimer interface. The coordination environment of K^+^ in the ion channels is described in detail in Supplementary Figure S10. In the TET26-1 monomer, the K^+^ ions in the middle and at the 3′-end are positioned nearly equidistantly from the two G-tetrads with an average K–O distance of 2.8 ± 0.1 Å. The K^+^ at the 5′-end is situated much closer to the 5′ G-tetrad with an average K–O distance of 2.6 ± 0.1 Å compared to 3.1 ± 0.3 Å from the middle tetrad. We can explain this abnormality in two ways. It is possible that the metal in this position is, in fact, Na^+^ or a combination of Na^+^ and K^+^ (each with a partial occupancy) because the annealing buffer contained 10 mM sodium cacodylate and the crystallization condition contained 300 mM NaCl. Na^+^ tends to occupy the position within the terminal G-tetrad due to its smaller radius (21). However, our attempts to replace K^+^ with Na^+^ or to split it into two ions during structure refinement did not improve the model. Verifying the metal identity with CheckMyMetal (33) suggested K^+^ as the more probable cation. Alternatively, the metal in this position could be a K^+^ that is drawn into the dimer interface toward the 5′ G-tetrad to compensate for the missing K^+^ at this position and aid the electrostatic stabilization of the dimer interface. The ASU of TET26-1 also contains a single sodium ion in an octahedral environment of four water molecules, the phosphate of G17, and the O2′ of T3′, Supplementary Figure S10B. This Na^+^ ion likely plays a role in stabilizing the crystal packing.

In TET26-2, all but the 3′-end K^+^ are equidistant from the G-tetrads with an average K–O distance of 2.7 ± 0.1 Å. The 3′-end K^+^ ion is located closer to the terminal tetrad with an average K–O distance of 2.63 ± 0.03 Å compared to 2.92 ± 0.02 Å from the middle tetrad. This is likely due to the relatively high non-planar deformation of the 3′-tetrad (*D*_OOP_= 1.83 Å, see Supplementary Table S9) which leads to a larger opening in its center. All K^+^–K^+^ distances are in the range of 3.22–3.31 Å. The ion channel is extended in the 3′ direction by a water molecule located 3.19 Å away from the 3′-end K^+^, Supplementary Figure S10.

In TET26-3, the coordination of K^+^ resembles that in Tel26-2, although the spread in values is more significant. All but the 3′-end K^+^ display an average K-O distance of 2.7–2.9 Å. The 3′-end K^+^ ion is located closer to the terminal tetrad with an average K–O distance of 2.52 ± 0.08 Å compared to 3.41 ± 0.07 Å from the middle tetrad. This unusually elongated K–O distance is accompanied by a longer than usual K^+^–K^+^ distance of 3.78 Å as compared to other K^+^–K^+^ distances in this structure, 3.34 and 3.43 Å. It is possible that this position is occupied by Na^+^ or by a combination of Na^+^ and K^+^ since Na^+^ is present in the crystallization condition. However, substitution of the K^+^ with Na^+^ led to an increase in *R*_free_.

### Structural parameters for TET26

*B-factors*. The overall B-factor values increase in the order TET26-1 < TET26-2 < TET26-3, Supplementary Figure S6B. As expected, the most ordered parts of all structures are the G-tetrads with B-factors of 43, 66, and 73 Å^2^ respectively. The loops and overhangs display a high degree of disorder with B-factors of 59, 97 and 129 for the loops and 80, 86 and 96 Å^2^ for the 5′-GTT overhangs in TET26-1, -2 and -3, respectively. Interestingly, the presence of K^+^ at the dimer interface in TET26-2 and -3 did not help to stabilize these GQs. The resolution of the three structures is nearly identical 1.97–2.00 Å. Solvent content differs between the three structures (29.1, 46.2 and 32.0%) but alone cannot explain the extent of the observed differences in the value of B-factors.

*Helical twist*. The average helical twist in all three TET26 structures is ∼30°, Supplementary Table S8. This value falls within the range 31 ± 3° reported for unimolecular parallel quadruplexes with propeller loops (48). The values of helical twists in TET26-2 and -3 have a large spread. For example, in TET26-2 the twist between tetrads 1–2, 38 ± 1°, is considerably higher than the twist between tetrads 2–3 and 3–4, which are 23.5 ± 0.4° and 25 ± 2°, respectively. This difference is potentially due to the involvement of tetrad 1 in the dimer interface.

*Distance between tetrads*. The average distance between the G-tetrads in all structures is ∼3.3 Å, Supplementary Table S9, in line with an ideal distance for an efficient π–π stacking (49,50).

*Out-of-plane deviation*. The 5′ G-tetrad at the dimer interface is highly planar (with *D*_OOP_ of 0.29–0.44 Å) and the deviations of the other G-tetrads increase with increasing distance from the dimer interface: *D*_OOP_ is 0.51–0.68 Å for tetrad 2, 0.82–0.94 Å for tetrad 3 and 1.41–1.83 Å for tetrad 4, Supplementary Table S9. The *D*_OOP_ of the 5′G-tetrad in TET26-1 (which does not form a lined-up dimer) is the highest among all three structures, 0.44 versus 0.29 and 0.32 Å for TET26-1, -2 and -3, respectively, confirming that dimer stacking increases the planarity of G-tetrads. The G-tetrad planarity trends observed in TET26 structures agree with trends seen for other GQ structures that exhibit dimerization. For example, Tel22-NMM (PDB ID: 4FXM) forms a 5′–5′ dimer of parallel GQs and has *D*_OOP_ values of 0.49, 1.08 and 1.89 Å for the 5′-, middle, and 3′ G-tetrad, respectively (51). Another example is the parallel dimeric GQ formed by (TGGGT)_4_-NMM (PDB ID: 6P45) which has *D*_OOP_ values of 0.39, 0.97 and 2.06 Å for the 5′-, middle, and 3′ G-tetrad, respectively (23). The similarity of *D*_OOP_ values likely reflect the intrinsic properties of 5′-5′ dimeric parallel GQs. This trend is rather different in TET25 which does not form a dimer and displays significant *D*_OOP_ values for both the 5′ and 3′ G-tetrads, Supplementary Table S9.

### Loops and overhangs in TET26

The parallel structure of TET26 GQ has three TT propeller loops. In TET26-1 the loops are well-defined while those in TET26-2 and -3 are significantly less ordered, Supplementary Figure S6B. The first thymine of each loop (T8, T14, T20) points into the solvent and the second thymine (T9, T15, T21) rests within the DNA groove as is also observed for the propeller loop in TET25 (see above), **T1**-NMM, **T7**-NMM (PDB ID: 6PNK and 6P45) (23), and in the NMR structure of (TTGGGG)_4_ in Na^+^ (PDB ID: 186D) (18). In TET26-1, unexpectedly, the residues with the highest B-factors in loops 1 and 3 are the thymines within the grooves (T9 and T21) and not the thymines that point into the solvent (T8 and T20). In addition, loop 2 in TET26-1 (T14–T15) is extremely well ordered and its B-factors are comparable to those of nearby G-tetrad guanines, Supplementary Figure S6B. The high order of T8, T14–T15, and T20 is due to the multiplicity of their interactions with three neighboring GQs shown in Supplementary Figure S12. The hydrogen bonding network starts at T14, which interacts with T8′ from a second symmetry generated GQ. This T8′ hydrogen-bonds with T8′ from a third GQ, which in turn hydrogen-bonds with T14′ from a fourth GQ, Supplementary Figure S12A and B. π–π stacking between T8′–T14–T20′ and T8′–T14′–T20′ (Supplementary Figure S12A and B) and a strong water network provide further stabilization. The multiplicity of interactions involving nucleotides of loop 2 could lead to the observed offset of the TET26-1 dimer. High flexibility of the loops in TET26-2 and -3 results from the scarcity of intermolecular interactions between their bases.

The 5′-GTT and 3′-T overhangs in TET26 are engaged in extensive intermolecular interactions important for crystal packing, Supplementary Figure S13. In TET26-1, 5′-G1-T2 π–π stack on a 3′ G-tetrad from a neighboring GQ, forming hydrogen bonds with its 3′-T26′ overhang. In addition, T3 π–π stacks with T2, Supplementary Figure S13A. Variations of this pattern repeat in TET26-2 and -3. In TET26-2, the three 5′-GTT bases π–π stack with one another and rest via G1 on the 3′-tetrad of a neighboring GQ, forming a single hydrogen bond with the 3′-T26′, Supplementary Figure S13B. TET26-3 is missing its 5′-G1 and thus uses its 5′-T2 to π–π stack onto the 3′-tetrad of a neighboring GQ where it engages in a hydrogen bond with the 3′-T26′; at the same time T3 π–π stacks onto T2, Supplementary Figure S13C. The 5′-overhangs in TET26-1 and TET26-3 are less extended out, allowing for tighter packing, lower water content (29.1 and 32.0%, respectively), and higher quality of the structure for TET26-1. The 5′-overhang in TET26-2 extends further out into the solvent, leading to a looser packing, higher water content (46.2%), and larger dimensions of the unit cell, Table 2 (as compared to TET26-3 that crystallizes in the same space group).

**Table 2.** Crystallographic statistics for TET26 and TET25

	TET25 (Outer Shell)	TET26-1	TET26-2	TET26-3
Resolution range, Å	93.1–1.56	64.92–1.993	59.020–1.970	53.25–1.99
Highest resolution shell, Å	1.65–1.56	2.10–1.993	2.02–1.97	2.05–1.99
Space group	*P*12_1_1	*P*3_1_21	*P*2_1_2_1_2	*P*2_1_2_1_2
Unit cell dimensions				
*a*, *b*, *c* (Å)	29.998, 92.916, 50.081	38.681, 38.681, 64.921	32.042, 39.11, 59.02	30.09, 36.474, 53.253
α, β, γ (°)	90, 99.621, 90	90, 90, 120	90, 90, 90	90, 90, 90
Unique reflections	37 652 (5351)	4116 (557)	5471 (381)	4313 (299)
Redundancy	6.6 (6.3)	5.8 (4.6)	6.3 (6.6)	8.9 (8.4)
Completeness (%)	98.0 (96.1)	99.1 (94.6)	97.9 (98.8)	99.6 (95.8)
I/sigma	15.4	11.1	7.7	12.0
*R*-merge	0.071	0.099	0.106	0.069
*R*_work_/*R*_free_ (%)	0.1536/0.1825	0.1974/0.2092	0.2467/0.2615	0.2143/0.2392
Number of atoms	2633	585	537	532
DNA	2208	551	524	523
Solvent	379	30	9	5
Potassium	12	3	4	4
Sodium	0	1	0	0
Magnesium	6	0	0	0
Spermine	28	0	0	0
Copies in ASU	4	1	1	1
Overall *B*-factor for ASU	39.73	51.94	74.16	87.11
RMS deviations				
Bond length (Å)	0.012	0.005	0.006	0.010
Bond angles (°)	1.360	0.72	0.756	1.045
PDB ID	6XT7	6W9P	7JKU	7LL0

**Table 3.** Average groove widths in TET25 and TET26

Groove between strands #-#	1–2, Å	2–3, Å	3–4, Å	4–1, Å
**TET25**	14.2 ± 0.8	14.3 ± 0.4	13.0 ± 0.2	15.6 ± 0.2
**TET26-1**	14.5 ± 0.1	14.6 ± 0.1	14.6 ± 0.2	14.7 ± 0.2
**TET26-2**	14.4 ± 0.1	14.6 ± 0.2	14.6 ± 0.1	14.6 ± 0.2
**TET26-3**	14.5 ± 0.2	14.6 ± 0.1	14.5 ± 0.2	14.7 ± 0.1

### Biological relevance of the TET crystal structures

In this work, sample concentration differed across different experiments with biophysical measurements (TDS, CD, and thermal melts) performed at low concentrations of ∼4 μM, PAGE performed at 100–200 μM, and crystallization performed at high concentrations of 1.0–1.5 mM. To determine the effect of concentration on the GQ fold, we collected CD, thermal melt, and PAGE data on TET25 and TET26 samples at varying concentrations of 5–600 μM, Supplementary Figure S14, being limited by the cuvette pathlength. The data show that the concentration has a minor effect on the equilibrium of GQ conformations in both TET25 and TET26 samples with only a small decrease in sample stability at 600 μM by ∼3°C in both cases. This observation, coupled with PAGE data on DNA crystals (Figure 2B), indicates that our crystal structures represent biologically relevant conformations that preferentially crystallized and are not the result of crystal packing forces.

## CONCLUSION

In this work, we designed and investigated nine telomeric variants from *T. thermophila*. The basic structural motif of *T. thermophila* telomeric DNA, (GGGGTT)_3_GGGG (here TET22), adopts an antiparallel fold and contains a small amount of a (likely parallel) dimer due to an exposed 5′-G stretch. Addition of 5′ and/or 3′ overhangs to TET22 leads to a greater diversity of GQ conformations (two or even three) and an increase in the parallel component of their fold. The folding of one DNA sequence into multiple stable GQ conformations under the same solution conditions indicates a great plasticity of *T. thermophila* telomeric DNA. All four-tetrad GQs in this study display similar stability (*T*_1/2_ = 74–80°C) regardless of the overhangs, suggesting that the stability of *T. thermophila* telomeric GQs is controlled by the number of G-tetrads (and likely loop length).

While TET25 in solution exists as a mixture of three conformations, only the major hybrid [3 + 1] conformation crystallized. This structure displays a rare 5′-top snapback where the guanine from the 5′-GTT overhang participates in the G-tetrad formation, forcing one of the core guanines into a GTT loop. Such an unusual feature also leads to four loops in place of the typical three. We have also succeeded in crystallizing TET26 that differs from TET25 by the presence of a 3′-T. TET26 in solution exists as an equal mixture of two GQ conformations. The crystal structure of TET26 indicates that the parallel conformation of this DNA crystallized where the GQ core is formed by the guanines from the four GGGG stretches connected by three TT propeller loops, while the guanine from the 5′-GTT overhang participates in intermolecular interactions.

A survey of the Protein Data Bank reveals the prevalence of three-tetrad GQs, followed by four, and then two (52). Out of 329 GQ structures, only 188 are unimolecular. Among the four-tetrad GQs that are of interest to the current work, the most abundant are tetramolecular GQs formed by dGGGG and dTGGGGT sequences, and bimolecular GQs formed by dGGGGTTTTGGGG from *Oxytrichia nova*. Biologically relevant unimolecular four-tetrad GQs are rare. Several examples of right- and left-handed unimolecular four-tetrad parallel GQs were reported by the Phan lab. All are derivatives of the AGRO100 aptamer with irregularly spaced GG tracts and isolated Gs (53–57). These structures are composed of a dimer of linked two-tetrad GQs in place of a continuous four-tetrad topology. There are three reported unimolecular four-tetrad antiparallel GQs. One is an X-ray structure of a G-rich motif found in *Dictyostelium discoideum* published by our laboratory (24). The second example is the NMR structure of a GQ formed by the d(GGGGCC)_4_ sequence from *C9orf72* of the non-coding region involved in ALS/FTD neurodegenerative disorders published by the Plavec laboratory (58). The third example includes two NMR structures of GQs formed by dG_4_T_*x*_G_4_T_4_G_4_A_2_G_4_ (where *x* = 2 or 3) from the da Silva lab (59). To the best of our knowledge, the PDB does not contain right-handed unimolecular four-tetrad GQs of a parallel or hybrid [3 + 1] topology. Therefore, TET25 and TET26 are the first examples of such structures. Furthermore, TET25 contains a rare 5′-top snapback feature. As such, the results presented in this work greatly expand our knowledge about the diversity of GQ topologies. The coordinates reported here can be used to improve *in silico* drug screening and GQ-fold predicting algorithms to include topologies and unique structural features which have not been experimentally observed before.

## DATA AVAILABILITY

TET25 structure was deposited into PDB and assigned PDB ID: 6XT7. TET26-1, -2 and -3 structures were deposited into PDB and assigned PDB ID: 6W9P, 7JKU and 7LL0, respectively.

## Supplementary Material

gkac091_Supplemental_File
