# Supplementary material for: The first crystal structures of hybrid and parallel four-tetrad intramolecular G-quadruplexes
Source: Nucleic Acids Res. 2022 Feb 25;50(5):2959–72. doi: 10.1093/nar/gkac091 (PMC8934647; doi:10.1093/nar/gkac091)
Supplement: gkac091_Supplemental_File [file gkac091_supplemental_file.docx]

**Supporting Information**

**The First Crystal Structures of Hybrid and Parallel Four-Tetrad Intramolecular G-Quadruplexes**

Dana Beseiso, Erin V. Chen, Sawyer E. McCarthy, Kailey N. Martin, Elizabeth P. Gallagher, Joanne Miao, & Liliya A. Yatsunyk^*^

Department of Chemistry and Biochemistry, Swarthmore College, 500 College Ave, Swarthmore, PA 19081, USA

*corresponding author lyatsun1@swarthmore.edu

**Table S1**

**Table S1:** Extinction coefficients and molecular weights for sequences under study.

| Sequence | 𝜀_260_, M^-1^cm^-1^ | MW, g/mol |
| --- | --- | --- |
| TET12 | 115,000 | 3788.5 |
| TET14 | 134,400 | 4421.9 |
| TET22 | 213,400 | 7030.5 |
| TET22A | 210,800 | 7005.5 |
| TET24 | 229,000 | 7638.9 |
| TET24A | 229,400 | 7638.9 |
| TET25 | 240,300 | 7968.1 |
| TET26 | 248,800 | 8272.3 |
| TET26A | 245,600 | 8247.3 |

**Table S2**

**Table S2.** RMSD values between the different TET25 chains. For chain B, an overlay of both conformations was used in the calculation.

| **Chains** | **RMSD Value (Å)** |
| --- | --- |
| A-B | 0.827 |
| A-C | 0.576 |
| A-D | 0.670 |
| B-C | 0.910 |
| B-D | 0.968 |
| C-D | 0.781 |
| Average | 0.789 ± 0.146 |

**Table S3**

**Table S3:** Average groove widths for chains A-D in TET25 structure. BA and BB are the alternate conformations for Chain B. Tetrad 1 is the closest to the 5’ end. Distances were measured from C3’ to C3’ between strands using ACS-G4 program (<http://tiny.cc/ascG4>).

.


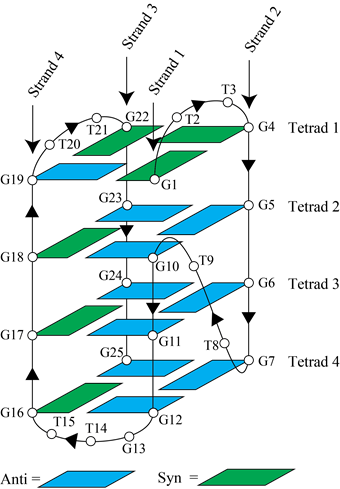


**Table S3A**: Average groove widths for GQ **chain A**.

| Grooves between strands #-# | | 1-2, Å | 2-3, Å | 3-4, Å | 4-1, Å |
| --- | --- | --- | --- | --- | --- |
| Chain A Tetrads | 1 | 12.9 | 14.0 | 12.8 | 15.4 |
|  | 2 | 14.4 | 14.4 | 13.1 | 15.8 |
|  | 3 | 14.7 | 14.5 | 13.0 | 15.5 |
|  | 4 | 14.6 | 14.5 | 12.9 | 15.6 |
| Average | | **14.1 ± 0.7** | **14.4 ± 0.2** | **12.9 ± 0.1** | **15.6 ± 0.1** |

**Table S3B**: Average groove widths for GQ **chain BA**.

| Groove between strands #-# | | 1-2, Å | 2-3, Å | 3-4, Å | 4-1, Å |
| --- | --- | --- | --- | --- | --- |
| Chain BA Tetrads | 1 | 13.0 | 13.9 | 12.9 | 15.4 |
|  | 2 | 14.6 | 14.4 | 13.2 | 15.7 |
|  | 3 | 14.8 | 14.4 | 12.8 | 15.5 |
|  | 4 | 14.5 | 15.1 | 12.8 | 15.5 |
| Average | | **14.2 ± 0.7** | **14.5 ± 0.4** | **12.9 ± 0.2** | **15.5 ± 0.1** |

**Table S3C**: Average groove widths for GQ **chain BB**.

| Groove between strands #-# | | 1-2, Å | 2-3, Å | 3-4, Å | 4-1, Å |
| --- | --- | --- | --- | --- | --- |
| Chain BB Tetrads | 1 | 13.0 | 13.9 | 12.9 | 15.4 |
|  | 2 | 14.6 | 14.4 | 13.2 | 15.8 |
|  | 3 | 14.8 | 14.4 | 12.8 | 15.5 |
|  | 4 | 14.6 | 13.7 | 12.8 | 15.5 |
| Average | | **14.3 ± 0.7** | **14.1 ± 0.3** | **12.9 ± 0.2** | **15.6 ± 0.2** |

**Table S3D**: Average groove widths for GQ **chain C**.

| Groove between strands #-# | | 1-2, Å | 2-3, Å | 3-4, Å | 4-1, Å |
| --- | --- | --- | --- | --- | --- |
| Chain C Tetrads | 1 | 12.8 | 13.8 | 12.8 | 15.5 |
|  | 2 | 14.3 | 14.3 | 13.2 | 15.7 |
|  | 3 | 14.6 | 14.5 | 13.3 | 15.5 |
|  | 4 | 14.7 | 14.4 | 12.8 | 15.5 |
| Average | | **14.1 ± 0.8** | **14.3 ± 0.3** | **13.0 ± 0.2** | **15.6 ± 0.1** |

**Table S3E**: Average groove widths for GQ **chain D**.

| Groove between strands #-# | | 1-2, Å | 2-3, Å | 3-4, Å | 4-1, Å |
| --- | --- | --- | --- | --- | --- |
| Chain D Tetrads | 1 | 13.0 | 13.8 | 12.9 | 15.3 |
|  | 2 | 14.5 | 14.2 | 13.2 | 15.7 |
|  | 3 | 14.9 | 14.5 | 13.1 | 15.6 |
|  | 4 | 14.9 | 14.5 | 12.7 | 15.7 |
| Average | | **14.3 ± 0.8** | **14.3 ± 0.3** | **13.0 ± 0.2** | **15.6 ± 0.2** |

**Table S3F**: Summary of the average groove widths for chains A-D.

| Groove between strands #-# | 1-2, Å | 2-3, Å | 3-4, Å | 4-1, Å |
| --- | --- | --- | --- | --- |
| Chain A | 14.1 ± 0.7 | 14.4 ± 0.2 | 12.9 ± 0.1 | 15.6 ± 0.1 |
| Chain B | 14.3 ± 0.7 | 14.3 ± 0.4 | 12.9 ± 0.2 | 15.6 ± 0.2 |
| Chain C | 14.1 ± 0.8 | 14.3 ± 0.3 | 13.0 ± 0.2 | 15.6 ± 0.1 |
| Chain D | 14.3 ± 0.8 | 14.3 ± 0.3 | 13.0 ± 0.2 | 15.6 ± 0.2 |
| Average | **14.2 ± 0.8** | **14.3 ± 0.4** | **13.0 ± 0.2** | **15.6 ± 0.2** |

**Table S4**

**Table S4:** Intramolecular helical twist (°) between each adjacent tetrad pair in TET25 structure.

| Helical twist between tetrads: | Chain A | Chain BA | Chain BB | Chain C | Chain D | Average |
| --- | --- | --- | --- | --- | --- | --- |
| 1 and 2 | 15.3 ± 0.9 | 15.2 ± 1.0 | 15.2 ± 0.9 | 16.2 ± 0.8 | 15.8 ± 0.8 | **15.6 ± 0.5** |
| 2 and 3 | 31.9 ± 0.4 | 32.0 ± 0.5 | 32.0 ± 0.3 | 32.4 ± 0.4 | 31.7 ± 0.6 | **32.0 ± 0.3** |
| 3 and 4 | 24.7 ± 0.4 | 25.2 ± 1.1 | 23.0 ± 1.4 | 24.7 ± 0.2 | 24.6 ± 0.4 | **24.5 ± 0.3** |

**Table S5**

**Table S5:** Out-of-plane deviations (D_OOP_) measured in Å for the TET25 Structure. BA and BB are the alternate conformations for Chain B. Tetrad 1 is the closest to the 5’ end.

|  | **Chain A** | **Chain BA** | **Chain BB** | **Chain C** | **Chain D** | **Average** |
| --- | --- | --- | --- | --- | --- | --- |
| **Tetrad 1** | 1.23 | 1.35 | 1.35 | 1.29 | 1.29 | **1.30 ± 0.05** |
| **Tetrad 2** | 1.06 | 1.13 | 1.09 | 1.14 | 1.01 | **1.09 ± 0.05** |
| **Tetrad 3** | 1.10 | 1.08 | 1.08 | 1.06 | 0.98 | **1.06 ± 0.05** |
| **Tetrad 4** | 1.93 | 1.87 | 1.82 | 2.09 | 1.91 | **1.92 ± 0.10** |

**Table S6**

**Table S6:** Distance between G-tetrads (in Å) in TET25. BA and BB are two alternate conformations for Chain B. Tetrad 1 is the closest to the 5’ end.

| **Distance between tetrads** | **Chain A** | **Chain BA** | **Chain BB** | **Chain C** | **Chain D** | **Average** |
| --- | --- | --- | --- | --- | --- | --- |
| **1 and 2** | 3.44 | 3.47 | 3.41 | 3.45 | 3.44 | **3.44 ± 0.02** |
| **2 and 3** | 3.40 | 3.37 | 3.43 | 3.39 | 3.39 | **3.40 ± 0.02** |
| **3 and 4** | 3.43 | 3.44 | 3.45 | 3.45 | 3.45 | **3.44 ± 0.01** |

**Table S7**

**Table S7**: Average groove widths in TET26-1 and TET26-2. G-tetrad 1 is the closest to the 5’ end. Strands 1-4 are labeled in the schematics. Distances were measured from C3’ to C3’ between strands using the program G4: Topology and Grooves (G4SC), courtesy of L. Mouawad.


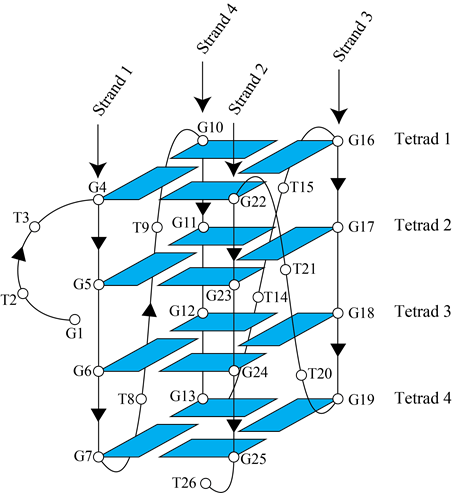


**Table S7A**: Average groove widths in **TET26-1**.

| Groove between strands #-# | | **1-2** | **2-3** | **3-4** | **4-1** |
| --- | --- | --- | --- | --- | --- |
| Tetrads | 1 | 14.4 | 14.4 | 14.3 | 15.1 |
|  | 2 | 14.6 | 14.6 | 14.5 | 14.7 |
|  | 3 | 14.5 | 14.7 | 14.8 | 14.5 |
|  | 4 | 14.5 | 14.7 | 14.8 | 14.6 |
| Average | | **14.5 ± 0.1** | **14.6 ± 0.1** | **14.6 ± 0.2** | **14.7 ± 0.2** |

**Table S7B**: Average groove widths in **TET26-2**.

| Groove between strands #-# | | **1-2** | **2-3** | **3-4** | **4-1** |
| --- | --- | --- | --- | --- | --- |
| Tetrads | 1 | 14.3 | 14.4 | 14.4 | 14.8 |
|  | 2 | 14.5 | 14.4 | 14.6 | 14.6 |
|  | 3 | 14.6 | 15.0 | 14.8 | 14.6 |
|  | 4 | 14.4 | 14.6 | 14.6 | 14.4 |
| Average | | **14.4 ± 0.1** | **14.6 ± 0.2** | **14.6 ± 0.1** | **14.6 ± 0.2** |

**Table S7C**: Average groove widths in **TET26-3**.

| Groove between strands #-# | | **1-2** | **2-3** | **3-4** | **4-1** |
| --- | --- | --- | --- | --- | --- |
| Tetrads | 1 | 14.27 | 14.62 | 14.21 | 14.63 |
|  | 2 | 14.64 | 14.55 | 14.62 | 14.59 |
|  | 3 | 14.75 | 14.71 | 14.73 | 14.83 |
|  | 4 | 14.42 | 14.56 | 14.56 | 14.78 |
| Average | | **14.5 ± 0.2** | **14.62 ± 0.08** | **14.5 ± 0.2** | **14.7 ± 0.1** |

**Table S8**

**Table S8:** Intramolecular helical twist (°) between each adjacent pair of G-tetrads in **TET26-1**, **TET26-2**, and **TET26-3** structures. G-tetrad 1 is [4,22,16,10]; G-tetrad 2 is [5,23,17,11]; G-tetrad 3 is [6,24,18,12]; and G-tetrad 4 is [7,25,19,13]. Strand 1 starts with G4, strand 2 starts with G22, strand 3 starts with G16, and strand 4 starts with G10.

| Between G-tetrads: | Strand | Helical Twist (^o^) in **TET26-1** | Helical Twist (^o^) in **TET26-2** | Helical Twist (^o^) in **TET26-3** |
| --- | --- | --- | --- | --- |
| 1 and 2 | 1 | 32.0 | 38.5 | 35.7 |
|  | 2 | 32.2 | 38.0 | 36.4 |
|  | 3 | 33.1 | 35.6 | 35.6 |
|  | 4 | 32.3 | 38.0 | 36 |
| **Average** |  | **32.4 ± 0.5** | **37.5 ± 1.3** | **35.9 ± 0.4** |
| 2 and 3 | 1 | 30.1 | 24.0 | 23.1 |
|  | 2 | 30.7 | 23.4 | 23.4 |
|  | 3 | 28.8 | 23.2 | 23.5 |
|  | 4 | 29.2 | 23.2 | 23.0 |
| **Average** |  | **29.7 ± 0.9** | **23.5 ± 0.4** | **23.3 ± 0.2** |
| 3 and 4 | 1 | 27.3 | 23.0 | 27.3 |
|  | 2 | 27.6 | 23.9 | 27.4 |
|  | 3 | 29.6 | 26.9 | 26.5 |
|  | 4 | 26.1 | 25.0 | 26.9 |

**Table S9**

**Table S9: D_OOP_ and distance between G-tetrads for TET25 and TET26 structures.** Tetrad 1 is the closest to the 5’ end.

|  | **D_OOP_ (Å)** | | | | **Distances between G-tetrads (Å)** | | |
| --- | --- | --- | --- | --- | --- | --- | --- |
|  | Tetrad 1, 5’-tetrad | Tetrad 2, middle | Tetrad 3, middle | Tetrad 4, 3’tetrad | Tetrad 1 → 2 | Tetrad 2 → 3 | Tetrad 3 → 4 |
| **TET25*** | 1.30 | 1.09 | 1.06 | 1.92 | 3.44 | 3.40 | 3.44 |
| **TET26-1** | 0.44 | 0.51 | 0.82 | 1.41 | 3.33 | 3.31 | 3.33 |
| **TET26-2** | 0.29 | 0.68 | 0.94 | 1.83 | 3.21 | 3.29 | 3.35 |
| **TET26-3** | 0.32 | 0.57 | 0.82 | 1.53 | 3.25 | 3.34 | 3.33 |
| **Tel22** | 0.49 | - | 1.08 | 1.89 |  |  |  |
| **T7** | 0.39 | - | 0.97 | 2.06 |  |  |  |

*the numbers come from Tables S5 and S6.

**
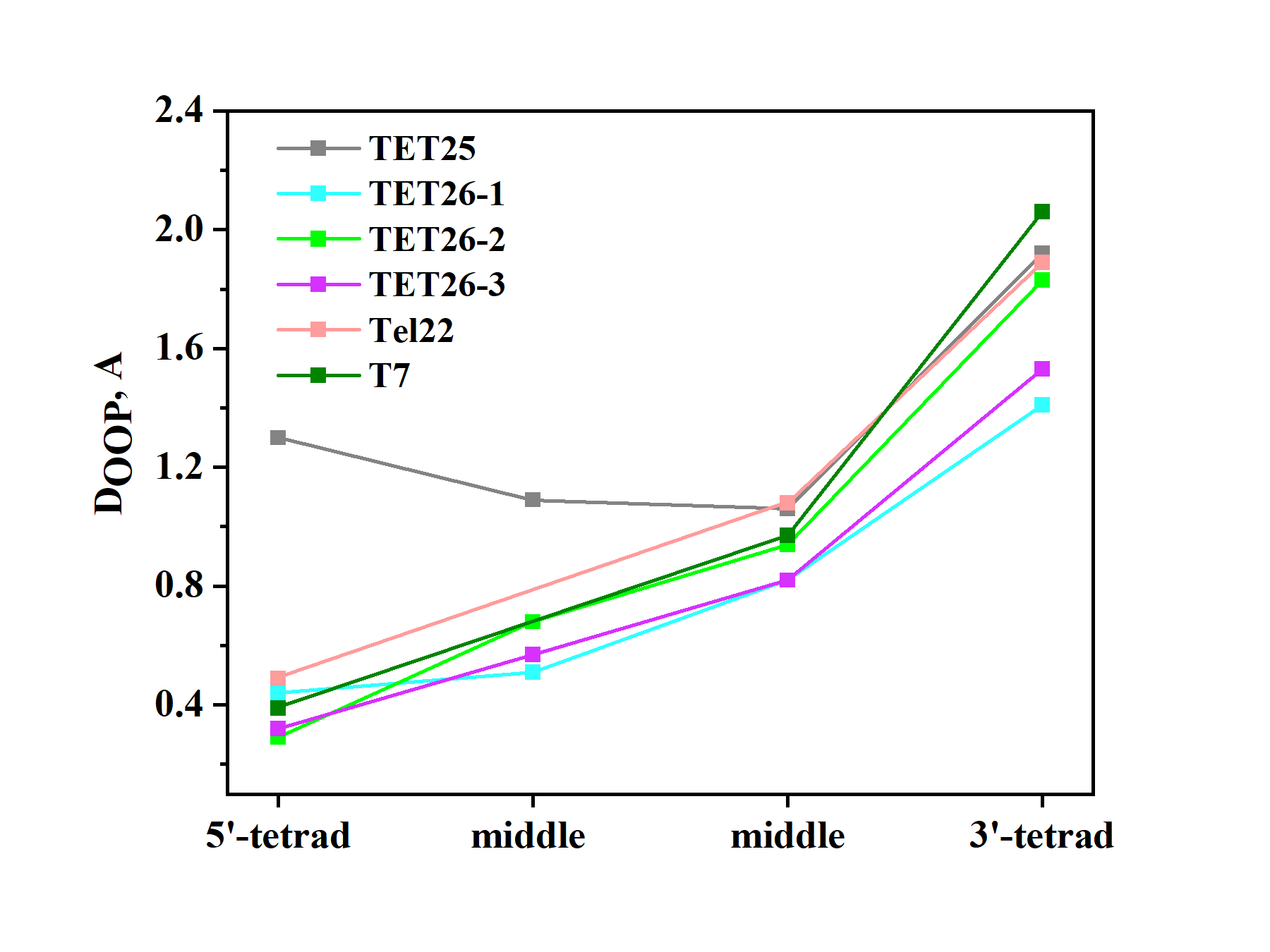
**

Tel22 has sequence AGGG(TTAGGG)_3_; T7 has a sequence (TGGGTT)_4_

**Figure S1**

**Figure S1**. *Folding of selected TET sequences as a function of buffer and annealing conditions.* (**A**) Fifteen percent gel was prepared in 1×TBE supplemented with 10 mM KCl. DNA samples were prepared in the following way: TET25 - DNA annealed in 10K buffer; TET25-LiOH - DNA treated with LiOH (see Materials and Methods for details). TET25-KCl - 10 mM KCl added during the annealing step. DNA concentration was 60-120 µM. Samples labeled +NMM contained 2 eq. of N-methylmesoporphyrin IX (NMM) added before the annealing step. (**B**) CD spectra of ~5 µM TET25, TET26, and TET26A alone and with 2 eq. of NMM in 10K buffer at 20 ℃. (**C**) CD scans for TET25 in crystallization condition and in 10K buffer at 20 ℃. Crystallization condition consists of 39% MPD, 0.165 M KCl, 0.02 M MgCl_2_, 0.04 M sodium cacodylate pH 6.5, and 0.012 M spermine tetrahydrochloride. Decrease in the parallel component is observed in the crystallization condition.


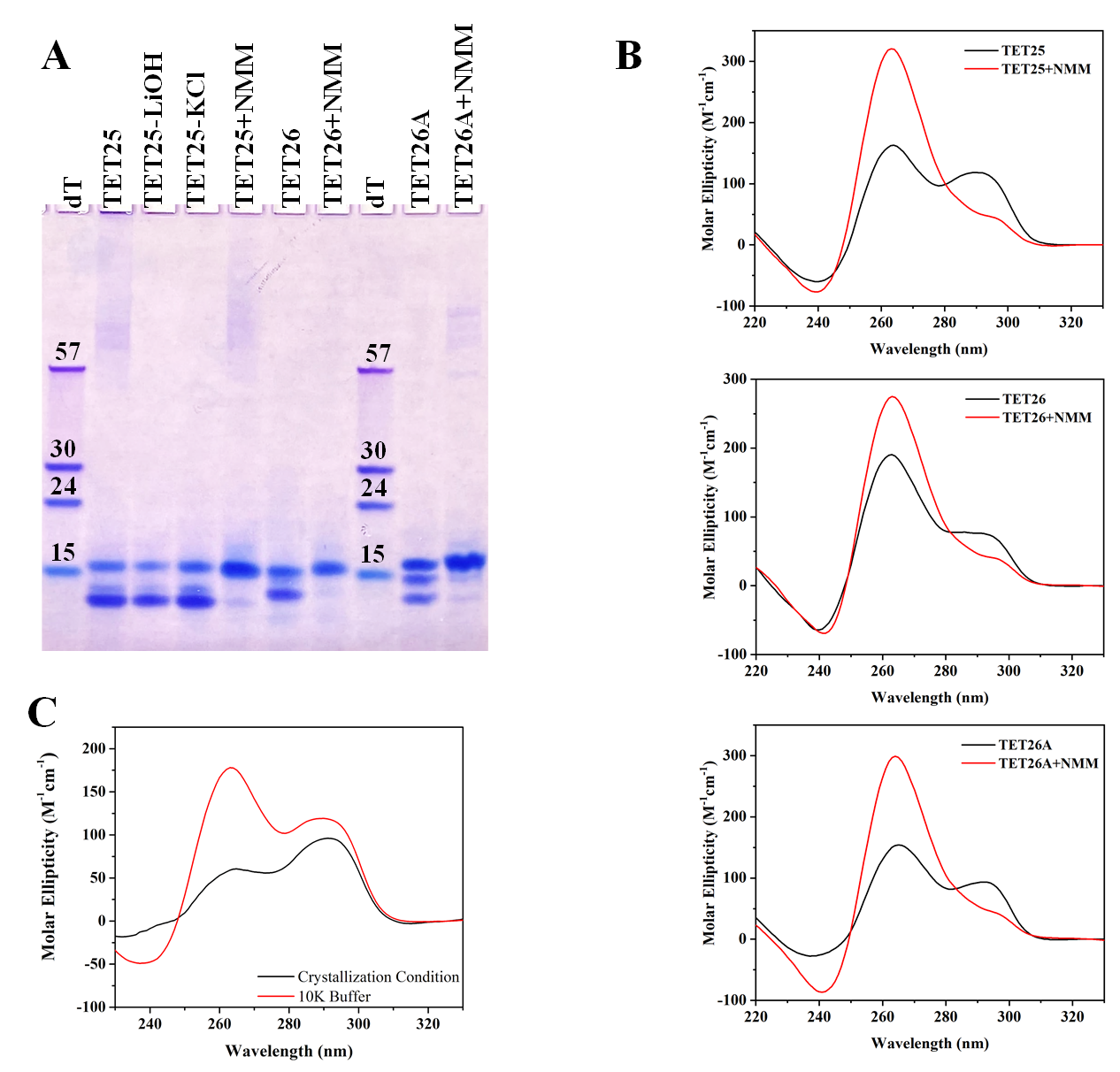


**Figure S2**

**Figure S2**: *Representative CD melting curves* for nine TET variants. DNA samples were prepared at ~4 µM per GQ in 10K buffer. CD melting data were collected at ~264 nm for all sequences but for TET22 and TET24A, for which data were collected at ~294 nm. We have collected CD scans before (see **Figure 1B-C** in the main text) and after the CD melting experiments to determine the reversibility of the melting process. Experiments were repeated 2-4 times.

**Figure S3**

**Figure S3**: *Crystal packing and representative crystal morphologies.* (**A**) Seven ASUs in TET25; (**B**) 18 ASUs in TET26-1, (**C**) 18 ASUs in TET26-2; (**D**) 18 ASUs in TET26-3 and (**E**) representative TET25 and TET26 crystals. The observed decrease in the unit cell dimensions for TEl26-3 as compared to Tel26-2 can be appreciated from comparison of (**C**) and (**D**).

**
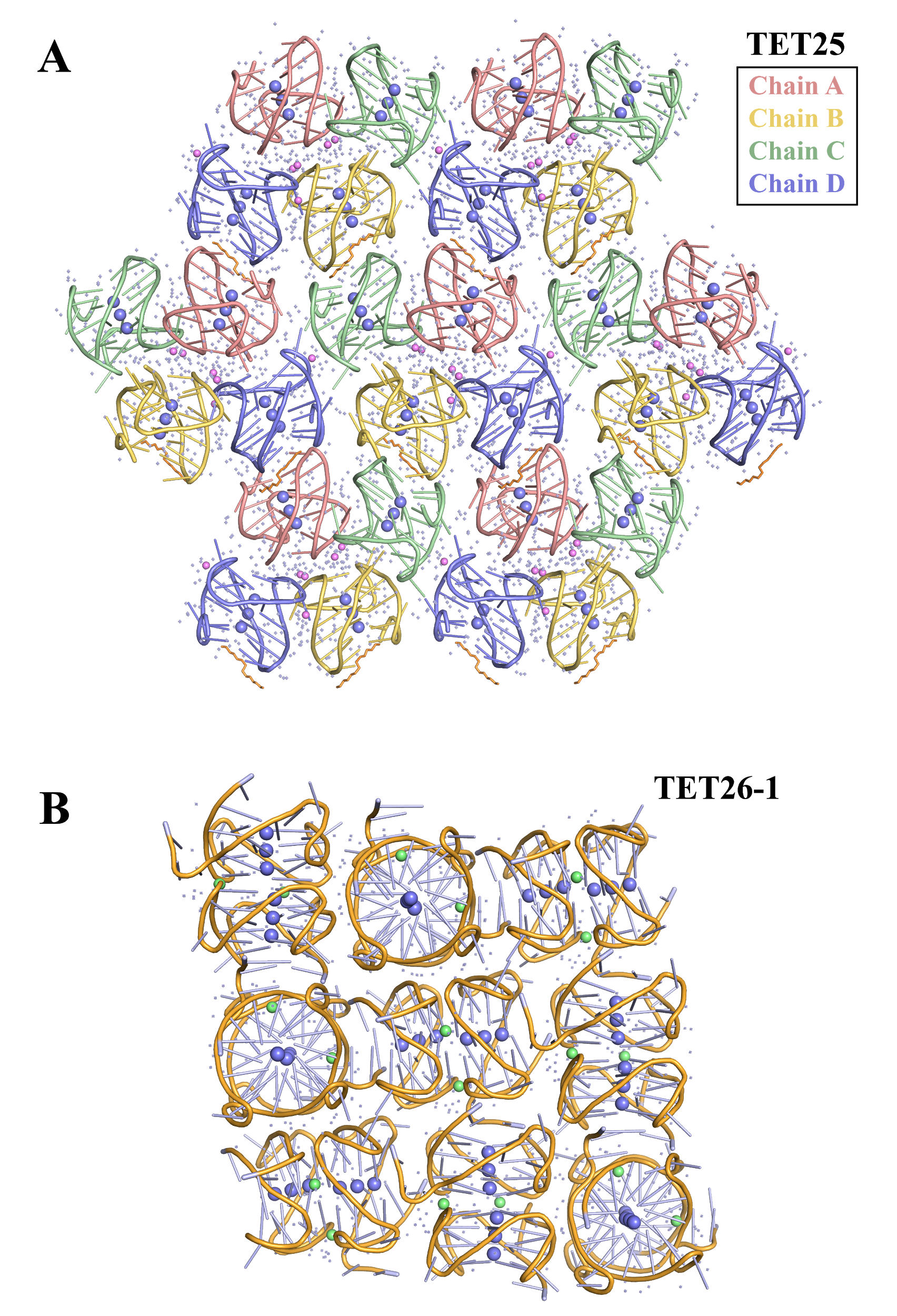
**

**
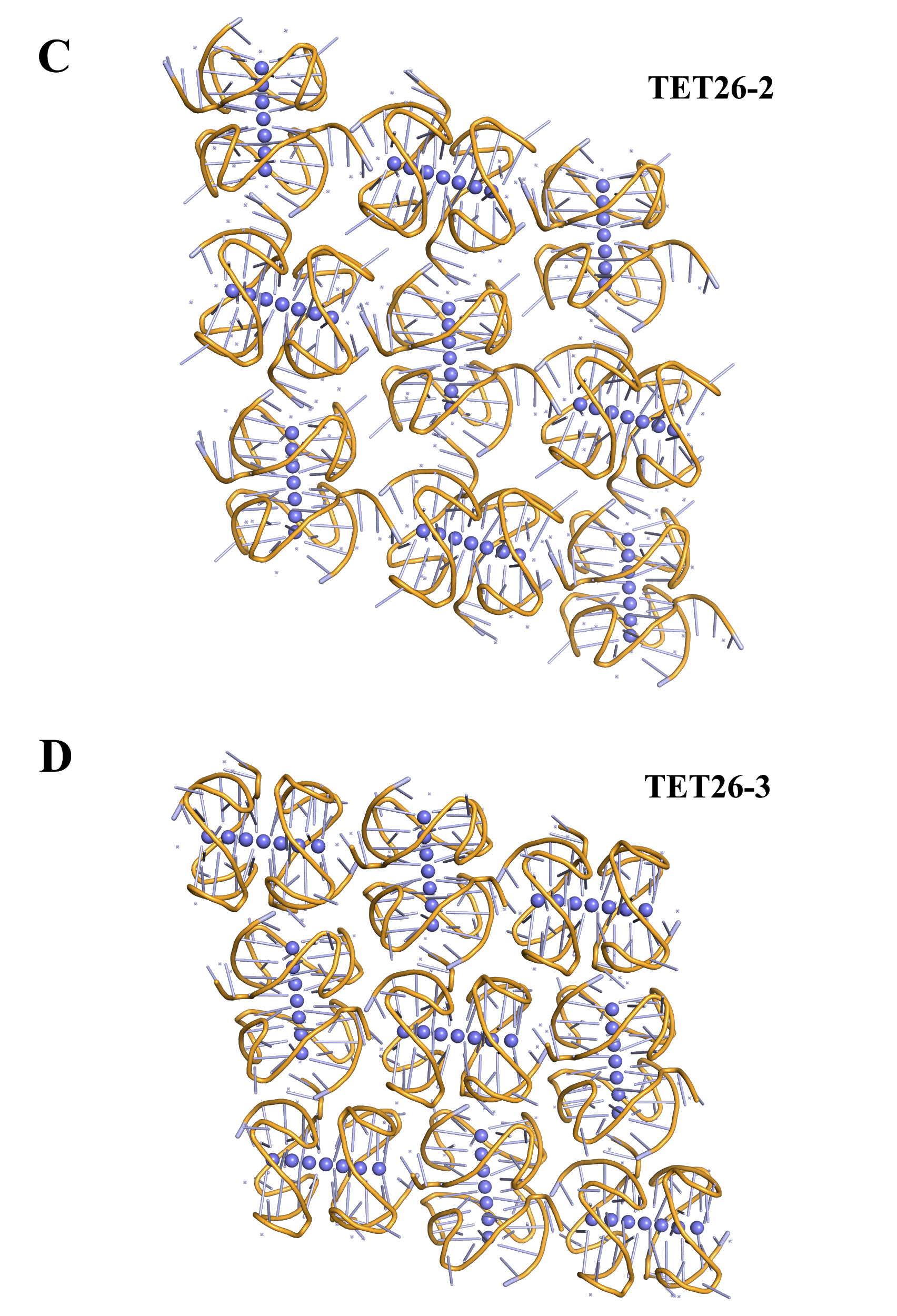
**

**Figure S4**

**Figure S4**: *Effect of overhangs on GQ fold.* CD data for selected sequences collected at 20 °C in a 10K buffer at ~4 µM per GQ. In all cases addition of overhangs leads to increase in parallel component of the CD signature. The same data, but in aggregate, is shown in **Figures 1B-C** of the main text.

**Figure S5**

**Figure S5**: *Asymmetric unit of TET25 structure and intermolecular interactions.* (**A**) Two different representations of the ASU. ASU contains four TET25 chains, A-D, three K^+^ ions per GQ depicted in blue (12 K^+^ total), two spermine molecules depicted in green (next to chains B and D); six Mg^2+^ depicted in green, and 379 water molecules depicted as small red spheres. (**B**) Coordination of Mg3-Mg6, which each coordinate four water molecules and two phosphates in *cis* arrangement. Specifically, **Mg3** bridges T14 phosphate of chain A and T14 phosphate of chain C; **Mg4** bridges T14 phosphates of chains D and B; **Mg5** bridges G17 phosphate of chain B and T9’ phosphate of chain B’; and **Mg6** bridges T9 and G7 phosphates from chain D. (**C**) Coordination sphere and interactions for Spermine 1, which bridges phosphates of chains B and C’. Spermine 2 (not shown) bridges phosphates of chains D and A’. All identified hydrogen bonds are strong-to-moderate with a distance between 2.6 - 3.2 Å.


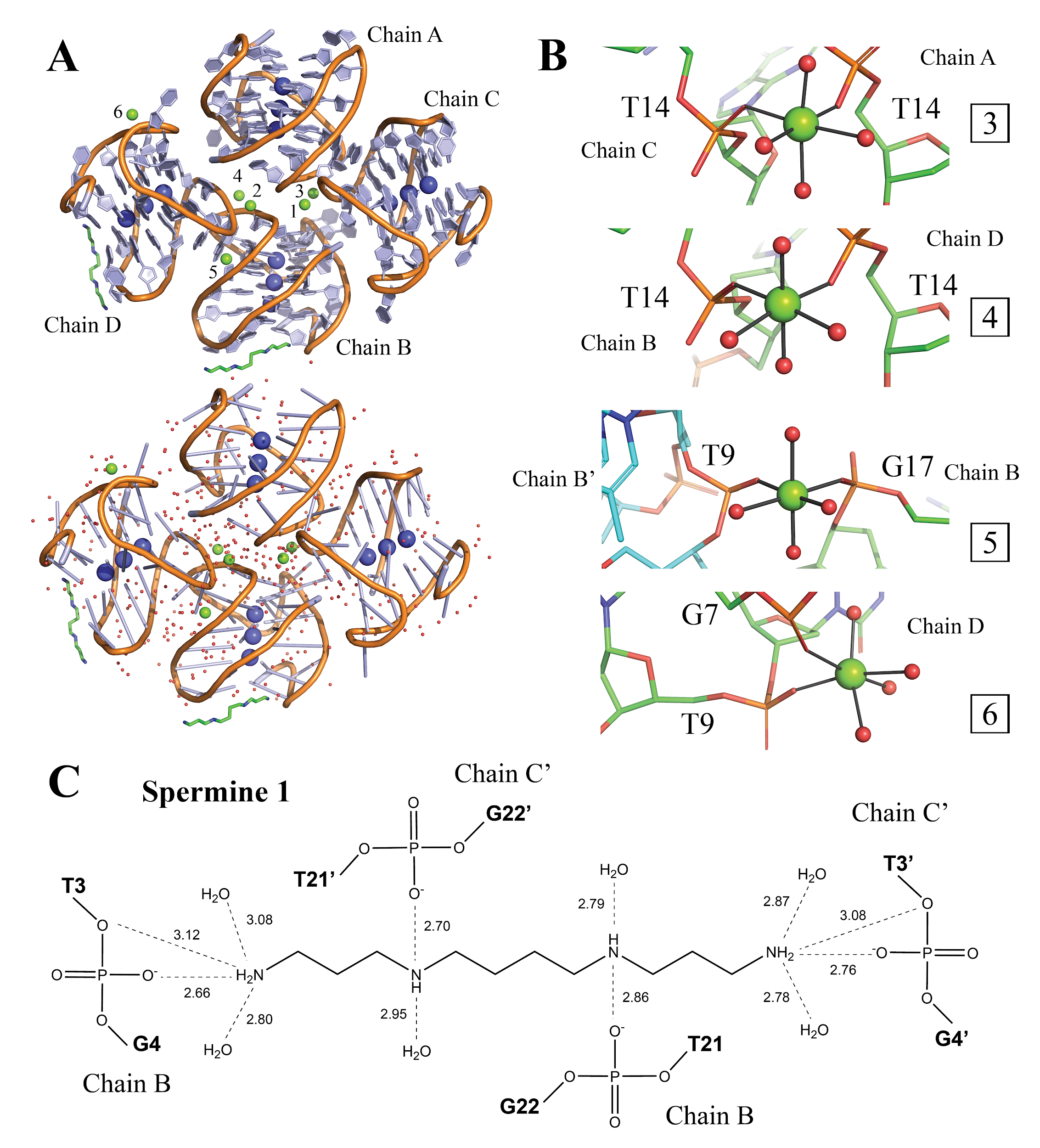


**Hydrogen bonding involving spermines.** The terminal nitrogens of each spermine are positioned above a GQ’s first tetrad, between strands 2 and 3. Each spermine is symmetric such that each half of it displays similar hydrogen bond patterns interacting with the same phosphates but in two different chains. Specifically, Spermine 1 has one end above chain B and the other above chain C’. Spermine 2 has ends above chains D and A’. The two spermines are similar, and Spermine 1 is shown above as a representative example. The secondary sphere interactions are intricate and extensive and likely further contribute to the bridging of the DNA chains and stabilizing this high-quality crystal lattice.

**Figure S6**

**Figure S6**: *Comparison of B-factors by nucleotide between GQ copies.* (**A**) Comparison between chains A-D in TET25 and (**B**) between TET26-1, -2, and -3. Note the significant difference in *y* axes for the two panels signifying superior quality of TET25 structure.


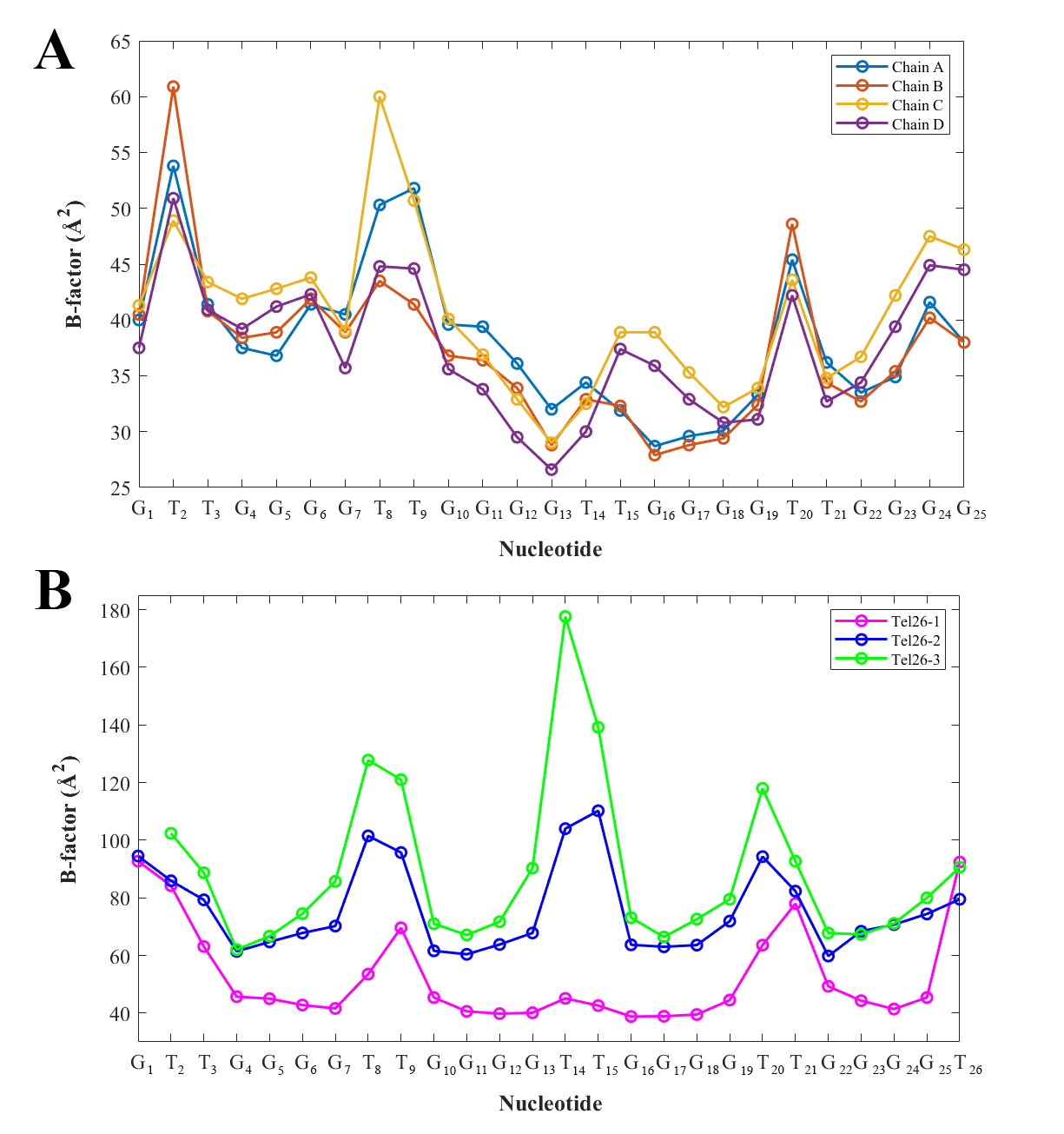


B-factors (Å^2^) for the TET25, TET26-1, TET26-2, and TET26-3 structures

|  | **TET25** | **TET26-1** | **TET26-2** | **TET26-3** |
| --- | --- | --- | --- | --- |
| **Overall for ASU** | 39.7 | 51.9 | 74.2 | 87.1 |
| **GQ with loops and overhangs** | 38.4 | 52.0 | 74.2 | 87.1 |
| **GQ core (four G-tetrads only)** | 37.1 | 42.7 | 65.8 | 72.9 |
| **Loops** | 40.9 | 58.7 | 96.8 | 128.6 |
| **5’-GTT overhang** | - | 79.8 | 86.4 | 95.5 |
| **3’-T overhang** | - | 92.4 | 79.5 | 90.6 |

**Figure S7**

**Figure S7**: *Groove width in TET25 and TET26*. (**A**) Average groove width for chains A-D in TET25. (**B**) Average groove width in TET26-1 - a representative example of TET26 structures. Average groove width values are indicated. Groove widths were measured using ACS-G4 program (<http://tiny.cc/ascG4>) and represent the distances between C3’-C3’ sugar atoms, as shown by dotted lines. Groove nucleotides are depicted as sticks. N, M, and W signify narrow, medium, and wide groove, respectively.


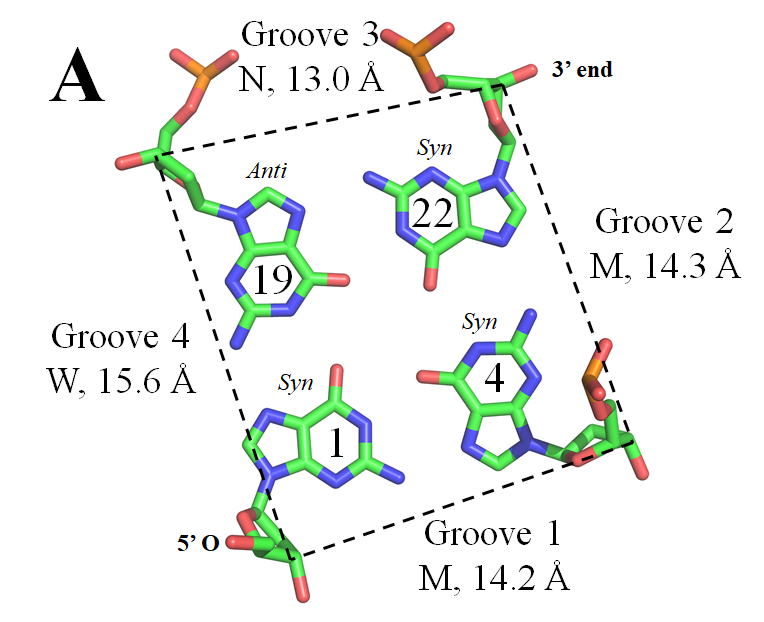


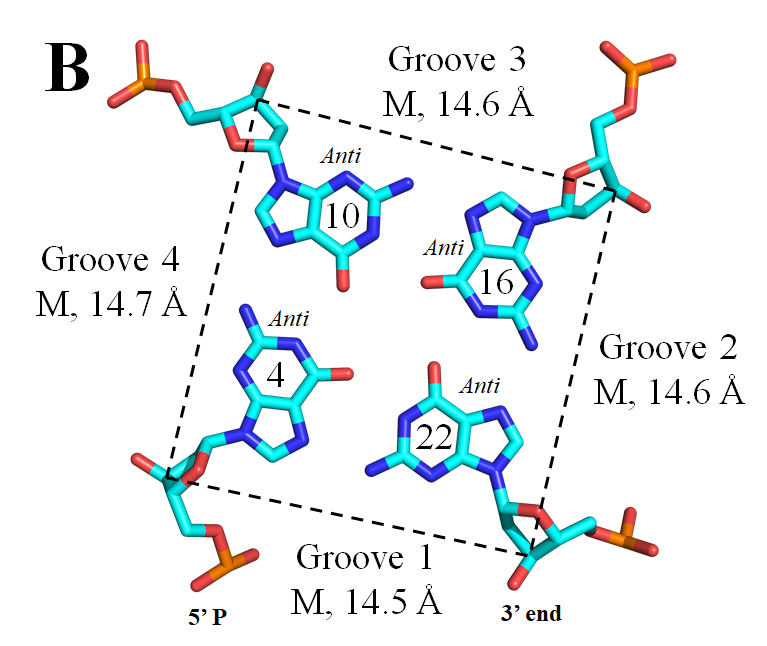


**Figure S8**

**Figure S8**: *Torsion angle wheel plots for* (**A**) chains A-D of TET25 (**B**) TET26-1, (**C**) TET26-2, and (**D**) TET26-3. Each individual angle is shown as a dot.


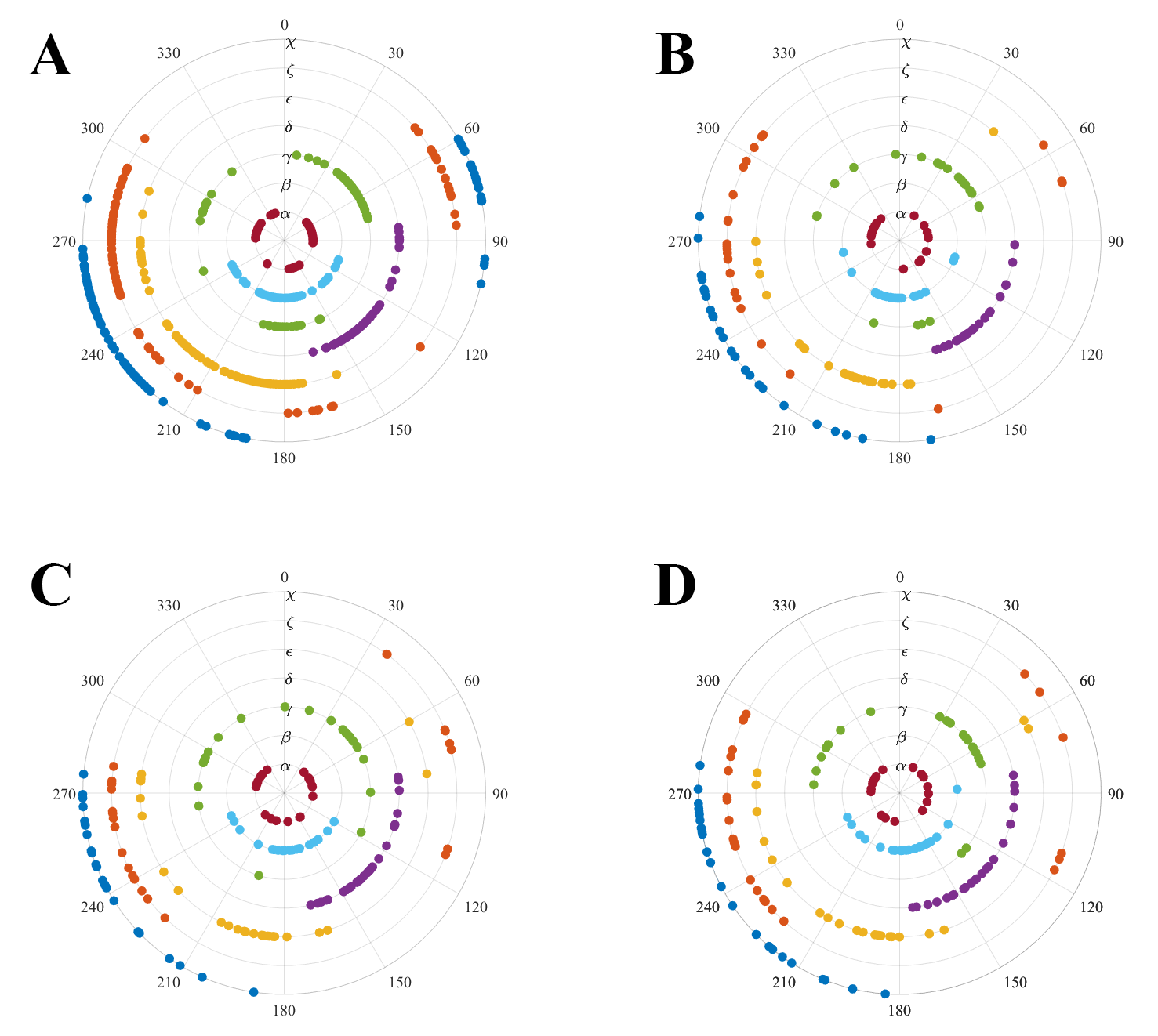


**Figure S9**

**Figure S9**: *Loop interactions in TET25*. (**A**) T8-T9 propellor loop with T8 pointing into the solvent and T9 tucked into a groove. (**B**) T3-T21 base pair stacked on top of the 5’ G-tetrad; formation of this base pair stabilizes two top lateral loops. (**C**) Interlocking G13-G13 base stack of Chains A and C; the same interaction occurs between G13s of Chain B and D. (**D**) Top and side view on T14 and T15 π-π stacking onto the 3’ G-tetrad, and (**E**) interaction between T20 and a symmetry generated T20’. Chain A was selected as a representative example. In **A**, **B**, and **D**, the tetrads are colored in blue while the loops are colored in orange. **
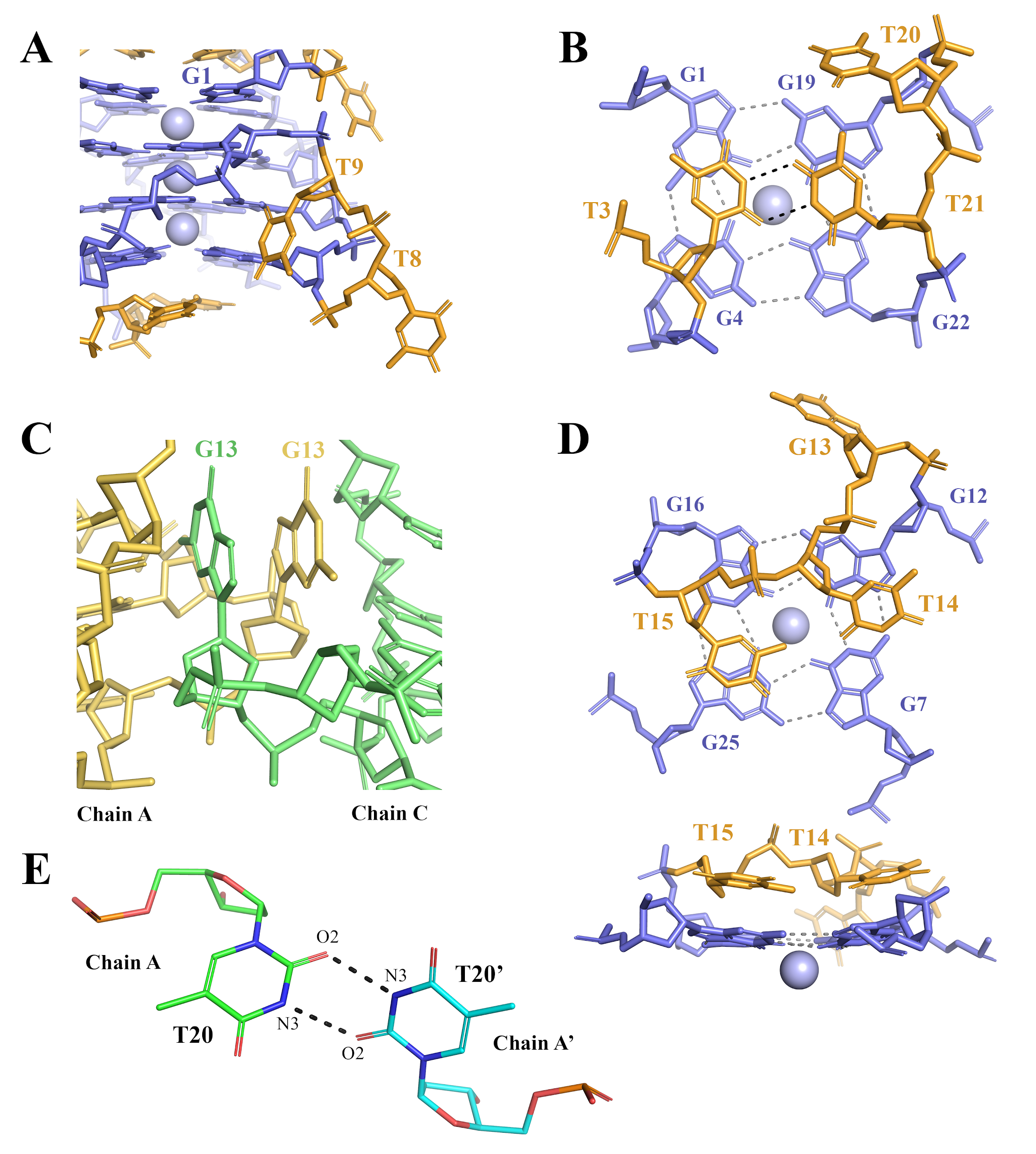
**

**Figure S10**

**Figure S10:** *Coordination of K^+^ and Na^+^ ions in TET25 and TET26*. (**A**) Schematic depiction of K^+^ ion channels with K-K and K-O distances marked. The values for TET25 represent average for Chains A-D. The K-O numbers refer to the average values for four K-O bonds. Oxygen atoms are depicted as red spheres. The top K^+^ in TET26-2 and TET26-3 represents K^+^ at the dimer interface. (**B**) Coordination of Na^+^ ion in TET26-1. Water molecules are depicted as small blue spheres. Figures were created using PyMol. All distances are shown in Å.


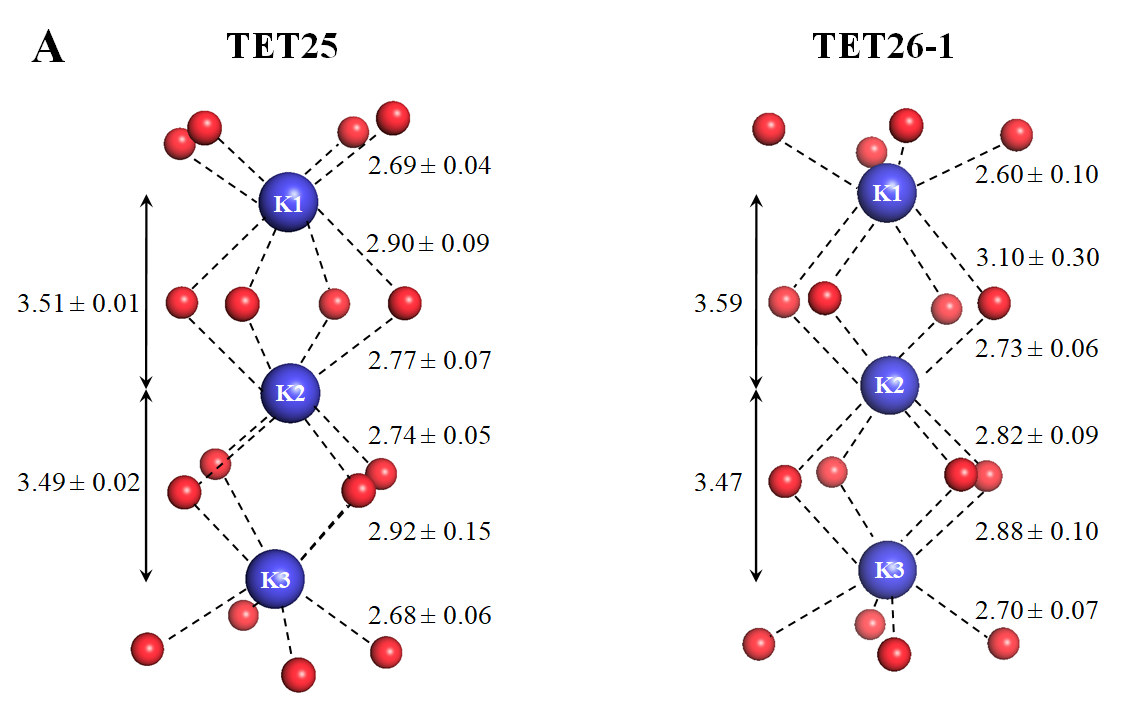

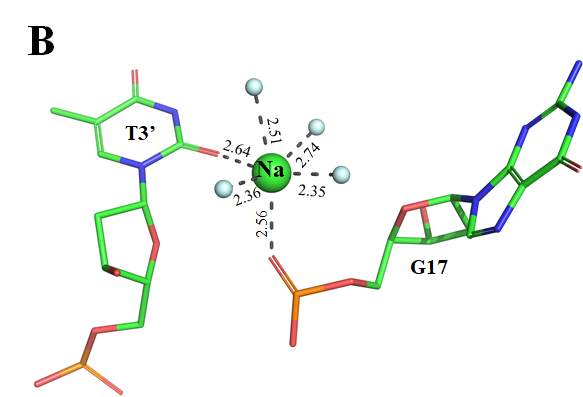


**Figure S11**

**Figure S11:** *Comparison of the three TET26 structures.* An overlay of the G-tetrad cores including K^+^ ions of TET26-1 (green), TET26-2 (pink), and TET26-3 (purple). The loops and overhang nucleotides were excluded from this alignment.


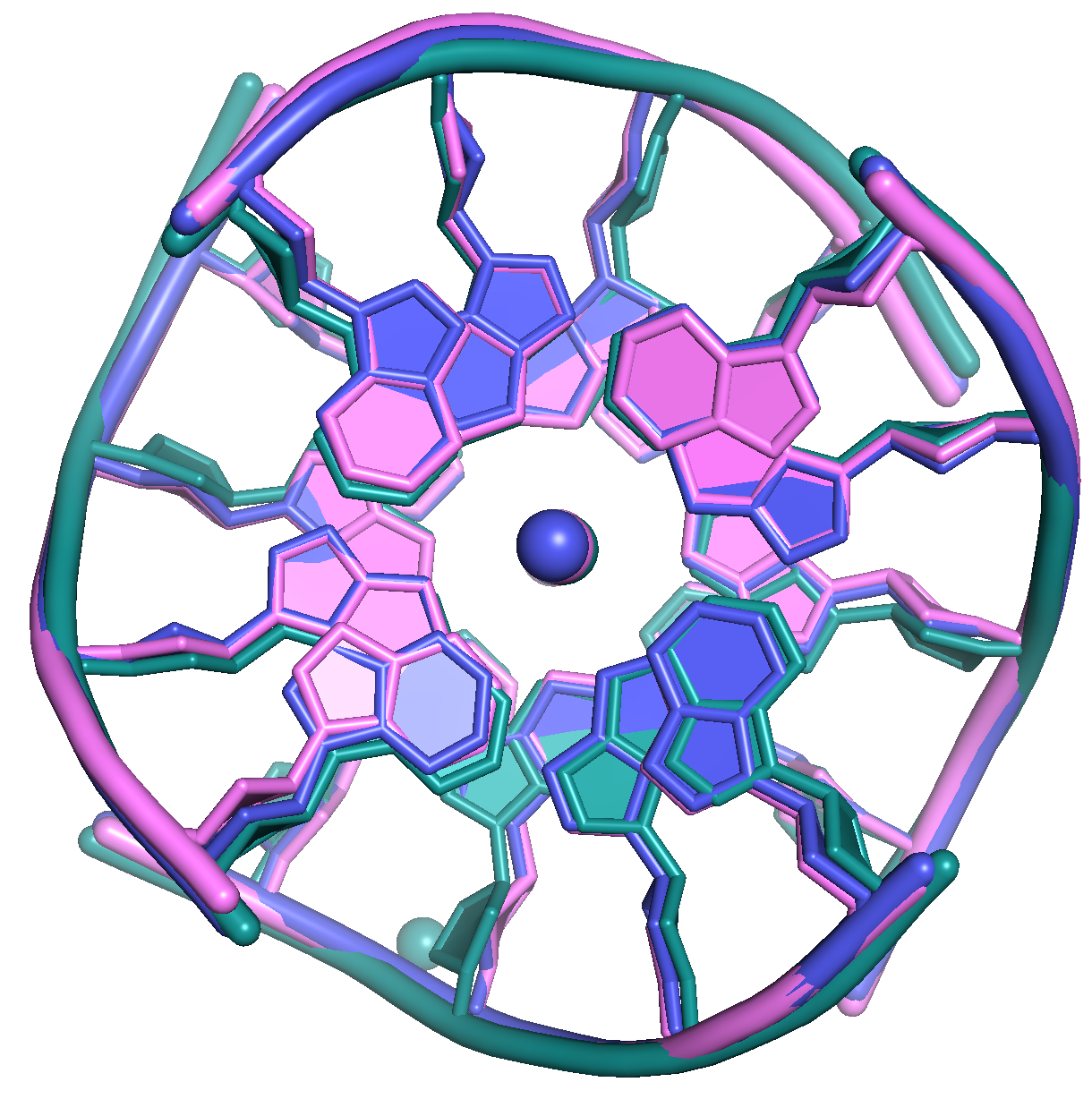


RMSD values calculated by aligning the TET26 structures in PyMOL. 1, 2, and 3 refer to TET26-1, TET26-2, and TET26-3, respectively.

| Full structures (Å) | | | Excluding the 5’ and 3’ overhangs (Å) | | | Excluding loops and overhangs (Å) | | |
| --- | --- | --- | --- | --- | --- | --- | --- | --- |
| 1 & 2 | 1 & 3 | 2 & 3 | 1 & 2 | 1 & 3 | 2 & 3 | 1 & 2 | 1 & 3 | 2 & 3 |
| 3.943 | 3.229 | 1.862 | 1.266 | 2.007 | 1.920 | 0.886 | 0.848 | 0.481 |

**Figure S12**

**Figure S12**: TT loop stability in TET26-1 (**A)** The hydrogen bonding network and π-π interactions between loop thymines. The T14, shown in yellow, is part of the ASU; all other nucleotides are symmetry generated. Each different chain is displayed in a different color. (**B**) ChemDraw representation of non-canonical base pairing between T14-T8’ and T8’-T8’ along with hydrogen bond distances in Å. (**C**) A zoomed-in side-view image of the *π-π* stacking shown in (**A)** between T8’, T14, and T20’. Distances were measured in PyMOL. The colors are the same as in (**A**).


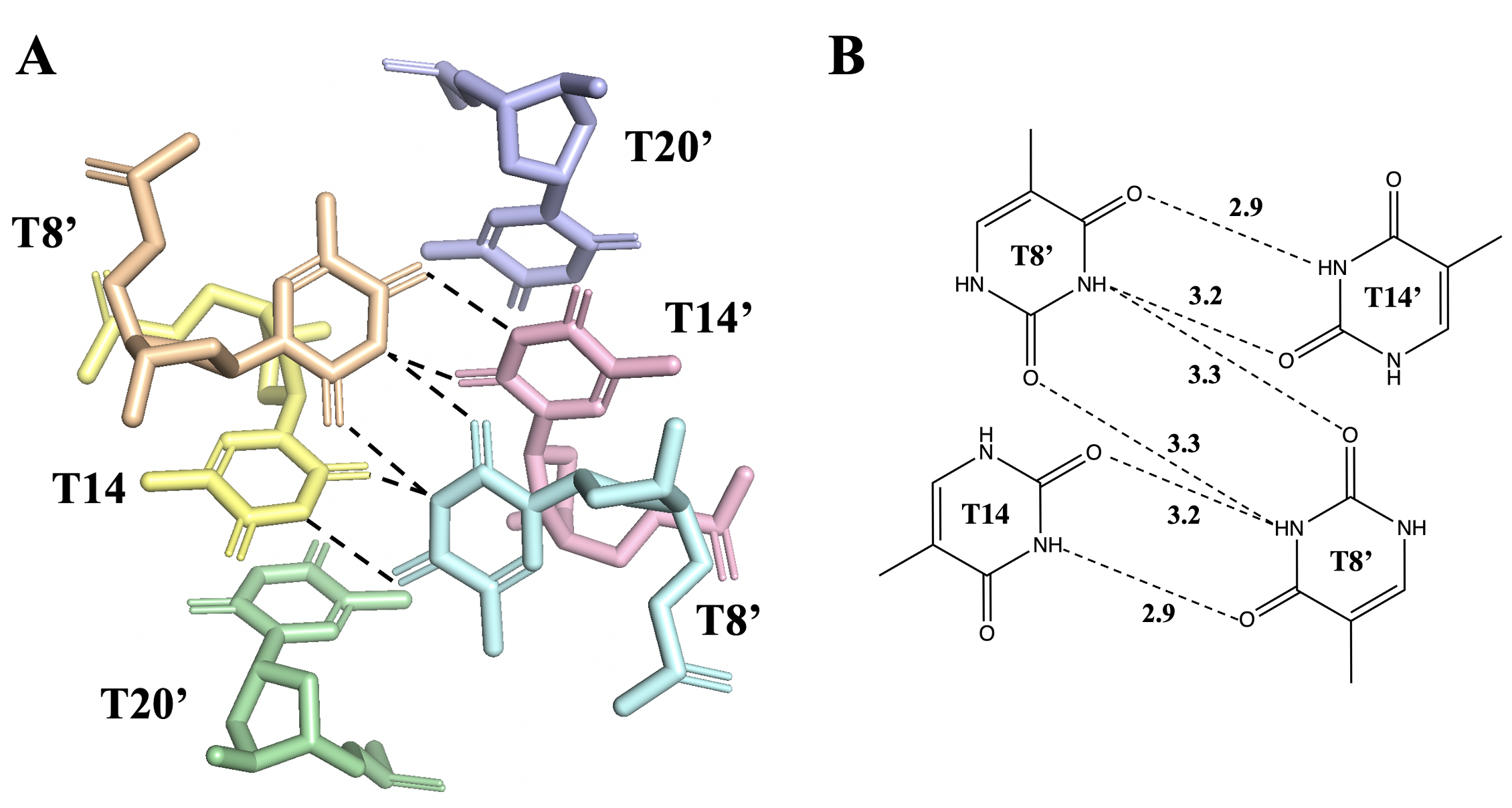


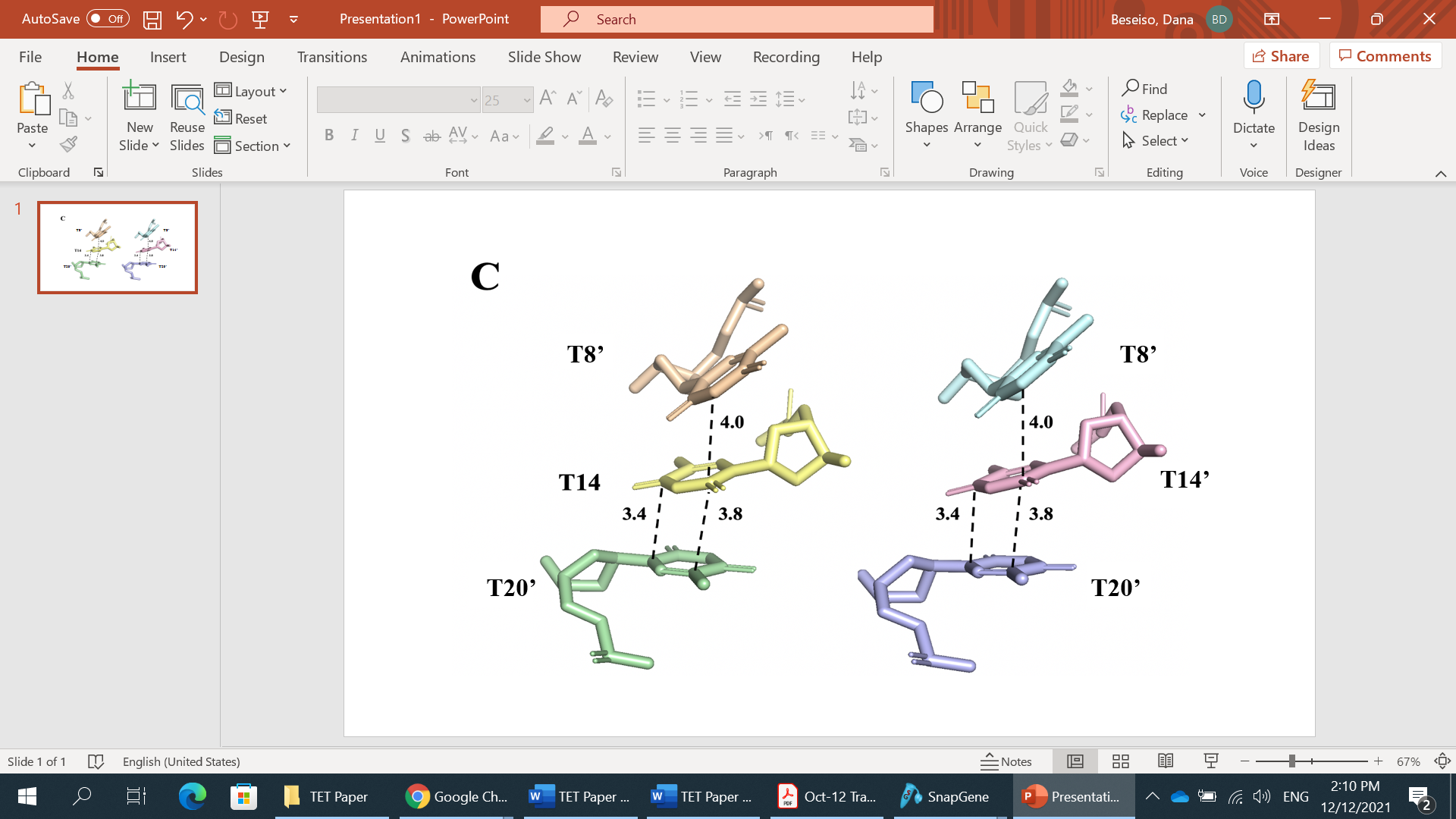


**Figure S13**

**Figure S13**: *The interactions of the 5’-GTT overhang in (****A****) TET26-1, (****B****) TET26-2, and (****C****) TET26-3 with symmetry generated GQs.* (Left) overall interactions; (center) zoom-in on the side view; and (right) zoom-in top view. The GQ in the ASU is colored in yellow and GQs generated by symmetry are colored in blue. The G1 nucleotide was not built in TET26-3.


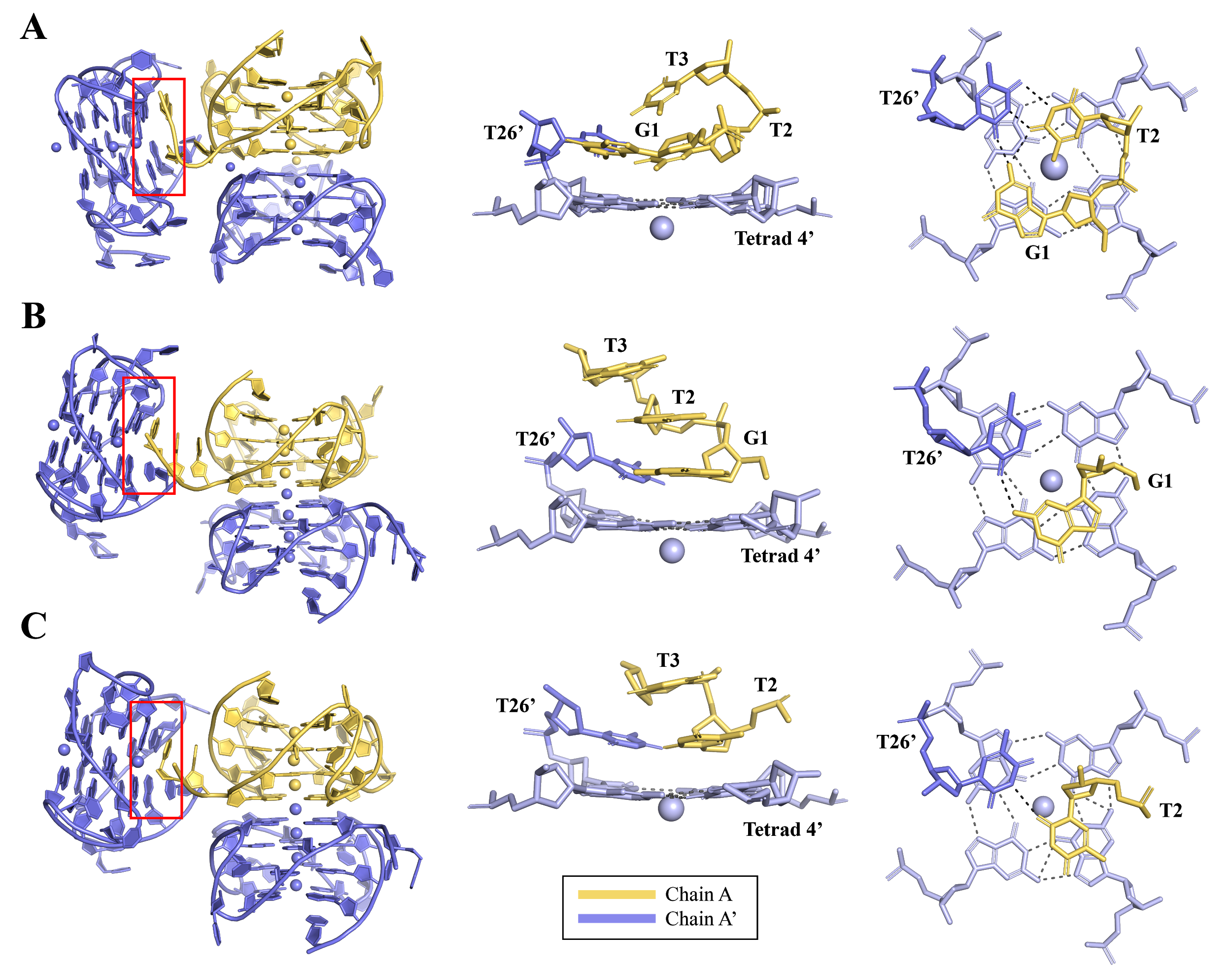


**Figure S14**

**Figure S14:** *The effect of DNA concentration on GQ conformation for TET25 and TET26.* **(A)** Fifteen percent gel was prepared in 1× TBE supplemented with 10 mM KCl. The DNA samples were prepared at concentrations of 5 – 600 µM and loaded such that each lane but that for 5 µM sample contained equal amount of DNA. (**B**) CD scans (left) and melting curves (right) for TET25. (**C**) CD scans (left) and melting curves (right) for TET26. (**D**) Thermodynamic parameters determined from melting curves. All experiments were conducted in 10K buffer.

**D**

| Concentration, μM | TET25 T_1/2_, °C | TET26 T_1/2_, °C |
| --- | --- | --- |
| 5 | 73.1 ± 0.3 | 70.9 ± 0.4 |
| 50 | 73.0 ± 0.3 | 71.2 ± 0.6 |
| 100 | 73.1 ± 0.3 | 71.1 ± 0.5 |
| 200 | 72.9 ± 1.4 | 70.6 ± 0.8 |
| 600 | 70.3 ± 0.3 | 67.8 ± 0.6 |

**small discrepancy with numbers presented in* ***Table 1*** *are due to the difference in the method used for T_1/2_ determination – CD melts (Table 1) vs UV-vis melts (here).*

**Figure S15**

**Figure S15**. Original Gel images for **Figure 2, Figure S1,** and **Figure S14**.

**
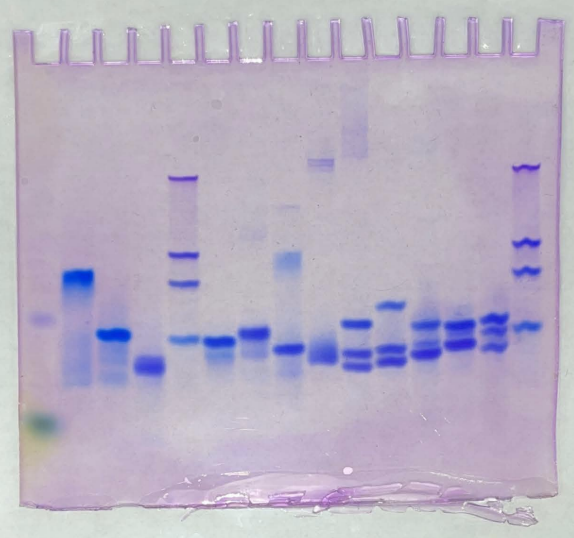
**

**
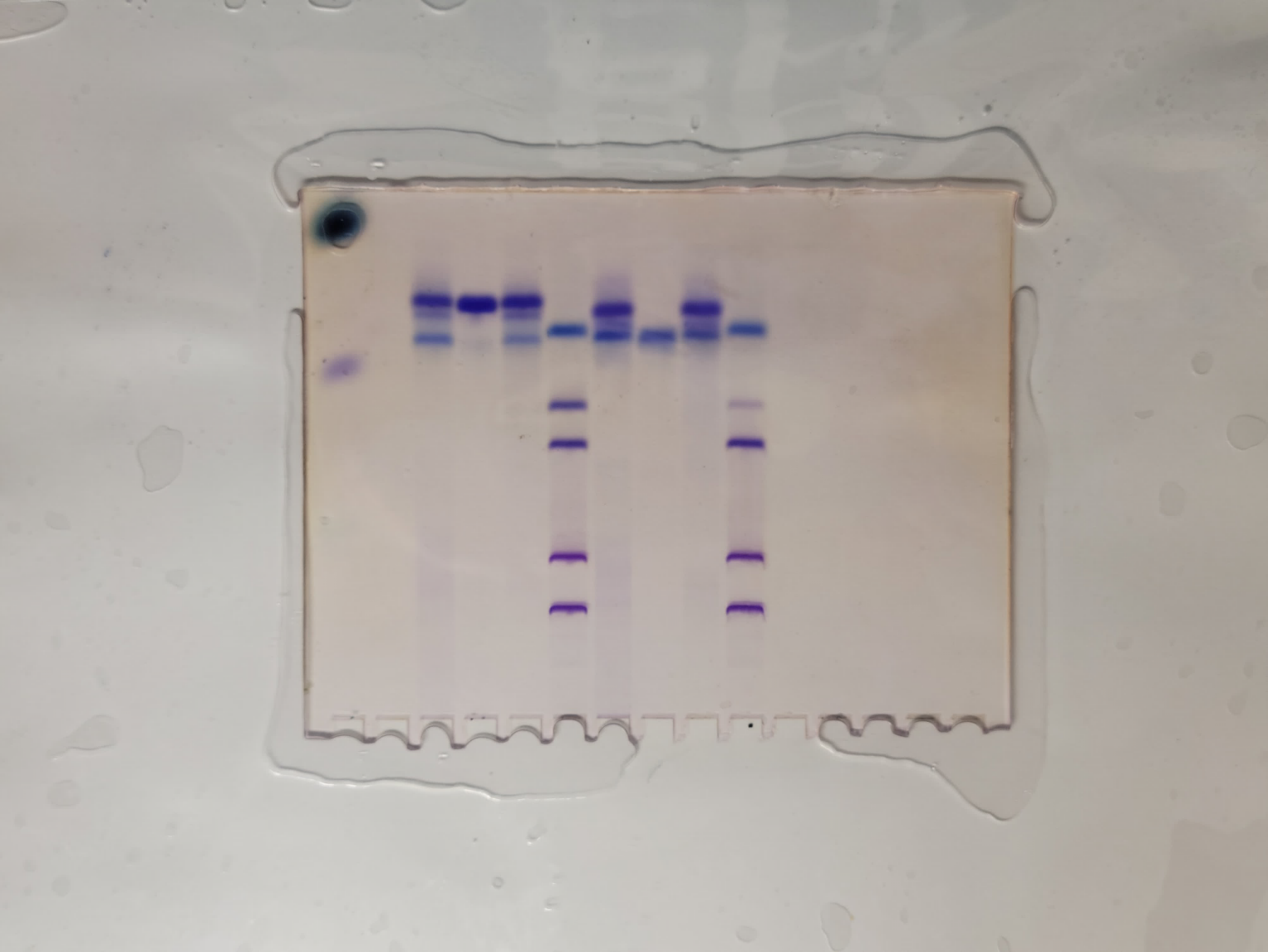
**

**
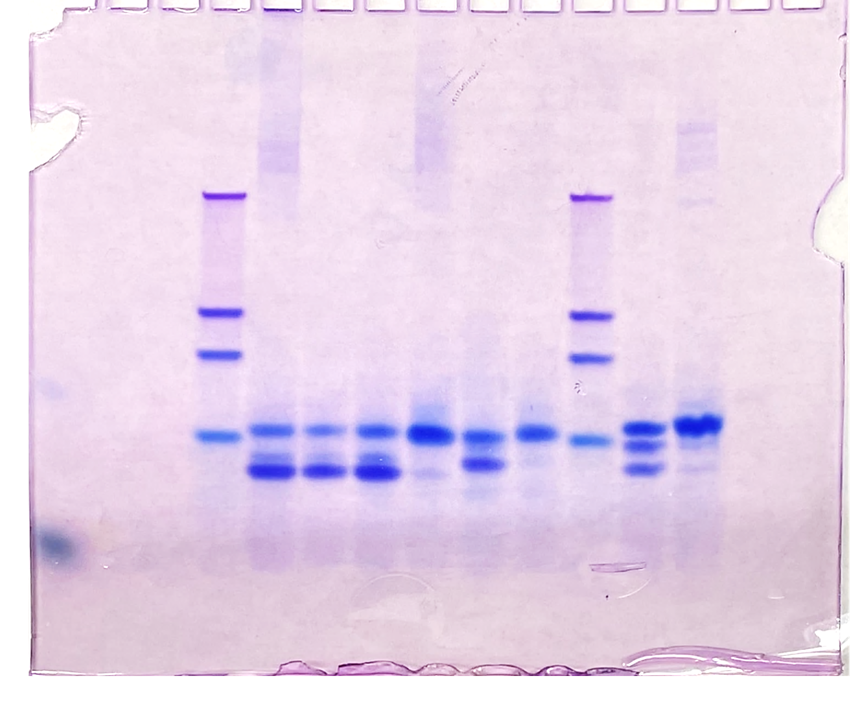
**

**
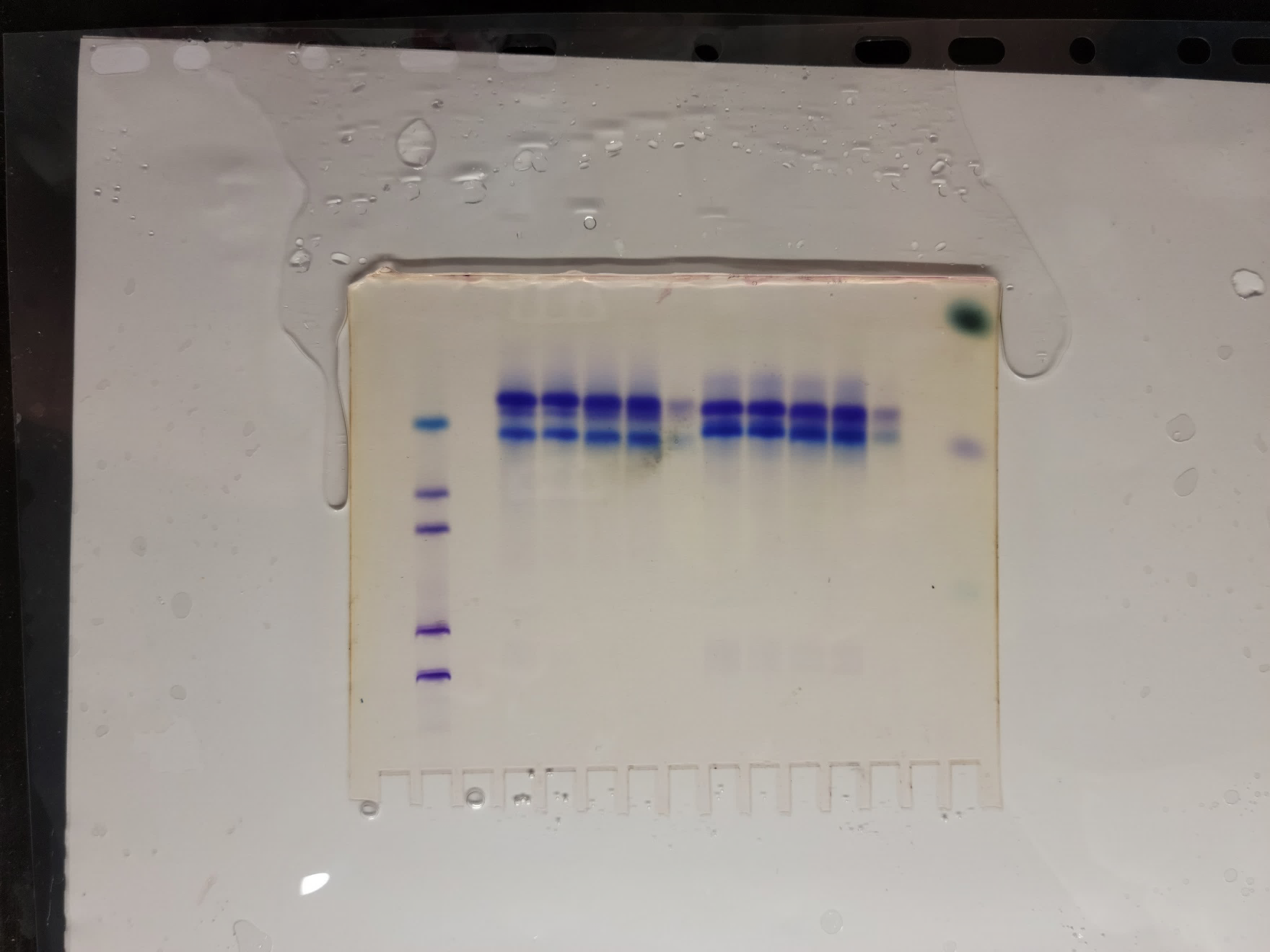
**
